# In vitro bioassays for monitoring drinking water quality of tap water, domestic filtration and bottled water

**DOI:** 10.1038/s41370-023-00566-6

**Published:** 2023-06-16

**Authors:** Beate I. Escher, Jordi Blanco, Josep Caixach, Dora Cserbik, Maria J. Farré, Cintia Flores, Maria König, Jungeun Lee, Jo Nyffeler, Carles Planas, Paula E. Redondo-Hasselerharm, Joaquim Rovira, Josep Sanchís, Marta Schuhmacher, Cristina M. Villanueva

**Affiliations:** 1 Helmholtz Centre for Environmental Research – UFZ, Department of Cell Toxicology, Leipzig, Germany; 2 Eberhard Karls University Tübingen, Environmental Toxicology, Department of Geosciences, Tübingen, Germany; 3 Laboratory of Toxicology and Environmental Health, School of Medicine, Universitat Rovira i Virgili, Reus, Spain; 4 Mass Spectrometry Laboratory/Organic Pollutants, Institute of Environmental Assessment and Water Research, IDAEA-CSIC, Barcelona, Spain; 5 ISGlobal, Barcelona, Spain; 6 Universitat Pompeu Fabra, UPF, Barcelona, Spain; 7 CIBER Epidemiología y Salud Pública, CIBERESP, Madrid, Spain; 8 Catalan Institute for Water Research, ICRA, Girona, Spain; 9 University of Girona, Girona, Spain; 10 Environmental Engineering Laboratory, Universitat Rovira i Virgili, Tarragona, Spain; 11 Hospital del Mar Medical Research Institute, IMIM, Barcelona, Spain; 12 Present Address: IMDEA Water, Madrid, Spain; 13 Present Address: Catalan Water Agency, Barcelona, Spain

**Keywords:** Water quality, Bioassay, Oxidative stress, Neurotoxicity, Disinfection by-products

## Abstract

**Background:**

Location-specific patterns of regulated and non-regulated disinfection byproducts (DBPs) were detected in tap water samples of the Barcelona Metropolitan Area. However, it remains unclear if the detected DBPs together with undetected DPBs and organic micropollutants can lead to mixture effects in drinking water.

**Objective:**

To evaluate the neurotoxicity, oxidative stress response and cytotoxicity of 42 tap water samples, 6 treated with activated carbon filters, 5 with reverse osmosis and 9 bottled waters. To compare the measured effects of the extracts with the mixture effects predicted from the detected concentrations and the relative effect potencies of the detected DBPs using the mixture model of concentration addition.

**Methods:**

Mixtures of organic chemicals in water samples were enriched by solid phase extraction and tested for cytotoxicity and neurite outgrowth inhibition in the neuronal cell line SH-SY5Y and for cytotoxicity and oxidative stress response in the AREc32 assay.

**Results:**

Unenriched water did not trigger neurotoxicity or cytotoxicity. After up to 500-fold enrichment, few extracts showed cytotoxicity. Disinfected water showed low neurotoxicity at 20- to 300-fold enrichment and oxidative stress response at 8- to 140-fold enrichment. Non-regulated non-volatile DBPs, particularly (brominated) haloacetonitriles dominated the predicted mixture effects of the detected chemicals and predicted effects agreed with the measured effects. By hierarchical clustering we identified strong geographical patterns in the types of DPBs and their association with effects. Activated carbon filters did not show a consistent reduction of effects but domestic reverse osmosis filters decreased the effect to that of bottled water.

**Impact statement:**

Bioassays are an important complement to chemical analysis of disinfection by-products (DBPs) in drinking water. Comparison of the measured oxidative stress response and mixture effects predicted from the detected chemicals and their relative effect potencies allowed the identification of the forcing agents for the mixture effects, which differed by location but were mainly non-regulated DBPs. This study demonstrates the relevance of non-regulated DBPs from a toxicological perspective. In vitro bioassays, in particular reporter gene assays for oxidative stress response that integrate different reactive toxicity pathways including genotoxicity, may therefore serve as sum parameters for drinking water quality assessment.

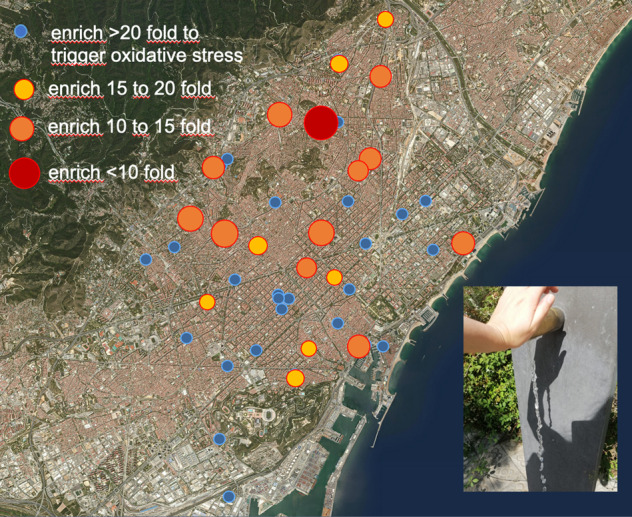

## Background

Water is a limited natural resource that is under pressure from human activity. The quality of our drinking water is threatened by the growing use of a wide range of chemicals that end up in the water cycle [1]. The scale of the challenge posed by drinking water contamination will increase in the future due to the increasing need for clean water, globally growing water scarcity due to climate change and a steep increase in the use of chemicals. We are all exposed to a cocktail of contaminants in drinking water [2] that may not be removed by standard treatments including pesticides, pharmaceuticals, personal care products (e.g., UV filters), ingredients from consumer products (e.g., poly- and perfluorinated compounds) and microplastics, or indeed are generated by drinking water production itself, e.g., distribution network materials, disinfection by-products (DBPs), and other transformation products [3].

While drinking water contaminants are present in mixtures [4], previous epidemiological research has focused on single chemicals or limited chemicals groups [5]. All chemicals contribute to mixture effects, even at low concentrations that, alone, might be below the threshold of effect [6, 7]. Animal studies suggest that the single-chemical paradigm underestimates cumulative effects of chemical mixtures [8]. The assessment of biological responses through in vitro assays has emerged as a useful tool to evaluate drinking water quality to provide insights into risks from (unknown) complex, low-level mixtures of micropollutants and DBPs [9].

In vitro bioassays have been widely applied in the past to assess drinking water quality in a comprehensive manner [9]. Source water can be contaminated with a wide range of organic micropollutants, which are typically reduced by drinking water treatment but depending on the treatment technology may still be present in drinking water, and with DBPs, which are formed during disinfection processes. Organic micropollutants and DBPs contribute to the mixture effects detected with in vitro bioassays. It is possible to differentiate between the contribution of micropollutants and DBPs to the measured mixture effect by measuring the in vitro effect of the SPE extract of water sampled directly before and after chlorination/disinfection [10]. Micropollutants cause diverse adverse outcome pathways among them endocrine disruption, reproduction toxicity, but also adaptive stress responses and carcinogenicity/mutagenicity. Accordingly diverse test batteries have been developed to capture the diversity of micropollutants’ toxicity (reviewed in [11]).

Most DBPs cause reactive toxicity, mainly oxidative stress response and genotoxicity/mutagenicity [12, 13]. The largest database of genotoxicity and cytotoxicity data on Chinese hamster ovary (CHO) cells dates back to 2000 [14] and has been further expanded over the years [15] and applied in numerous studies that tested drinking water [16, 17] and other water types [18]. Batteries of in vitro bioassays have been used to quantify different aspects of reactive toxicity pathways [9, 19]. The oxidative stress response quantified with a reporter gene assays indicative of the keap-nrf2-ARE pathway was shown to be a good measure of the specific effects of reactive DBPs, also because the soft electrophilic character of many DBPs lead only indirectly to genotoxicity [19]. As genotoxicity occurs often at similar or only slightly lower concentrations than cytotoxicity and cytotoxicity is a more integrative parameter where all chemicals and DBPs contribute to, albeit with different potency, water quality has also been assessed directly by cytotoxicity in CHO cells [20, 21].

DBPs have been reported to be neurotoxic [22] and activate the Nrf2-mediated oxidative stress response pathway [23] by reducing intracellular glutathione and increasing ROS [24]. Further there is some but not strong evidence that DBP exposure may adversely affect neuropsychological development [25]. Therefore, we also included for the first time a novel neurotoxicity assay based on the neurite outgrowth inhibition and cytotoxicity in differentiated neuronal SH-SY5Y cells [26, 27] in the evaluation of drinking water and extracts of drinking water.

Testing water samples directly in in vitro assays has often not resulted in detectable response. DPB mixtures in drinking water have to be enriched up to several hundred times to show measurable activity. For non-volatile DBPs, solid-phase extraction (SPE) has shown best recoveries [28, 29]. Volatile DBPs can be enriched with a purge-trap method but as the known volatile DBPs contribute only a minor fraction to the overall mixture effect with non-volatile DBPs dominating mixture toxicity [28], most studies to date applied SPE for sample preparation.

The Barcelona metropolitan area (BMA) exhibits unique and valuable characteristics to make it a suitable setting to conduct water studies. Drinking water is supplied from different sources (mainly Llobregat and Ter rivers), providing diverse water zones in a well-defined geographical area. This provides variability in the chemical concentration and composition, which allows to identify links with biological responses. In addition, the Llobregat river is intensively impacted by human activity, leading to the eventual occurrence of chemicals of industrial origin [30]. Previous studies in the area showed that levels of other drinking water contaminants e.g. arsenic were very low [31].

Regulated and non-regulated DBPs were measured in tap water samples collected at the 42 postal codes of the BMA [32]. DBPs were also quantified in tap water samples filtered with activated carbon (AC) and reverse osmosis (RO), as well as in bottled water [32]. Drinking water samples were analyzed for 11 haloacetic acids (HAAs), 4 trihalomethanes (THMs), 4 haloacetonitriles (HANs), 2 haloketones (HKs), chlorate, chlorite, and trichloronitromethane (TCNM). The median concentration of total THMs, HAAs and HANs, TCP, chlorite and chlorate in tap water were 42, 18, 3.2, 1.2, 53.9 and 214 μg/L, respectively. Chlorate, THMs, HAAs, and HANs were quantified in 98–100% tap water samples. Although both brominated and chlorinated DBPs were present; brominated species were found in a larger number of samples. AC filters reduced DBP levels in the range of 27–80%, and RO reduced DBP concentrations ≥ 98% [32]. In bottled water, only chlorate was detected in 3 out of 10 brands, with a median concentration of 13.0 µg/L [32].

## Objectives

The objective of this study was to complement the previous work on exposure to regulated and non-regulated DPB in the BMA [32], micropollutants [33] and micro(nano) plastic [34] by effect-based method that capture the entirety of mixture exposure of extracted micropollutants and DBPs with a focus on cytotoxicity, activation of oxidative stress response and neurotoxicity. Iceberg modelling, a specific form of mixture toxicity modelling, was used to identify mixture effect drivers for the endpoint of oxidative stress response.

AC filters and home RO systems have become quite popular for polishing tap water at the home. Previous studies have demonstrated the variable removal efficacy of effects measured with in vitro bioassays by point-of-use filters, ranging from 25 to 100% depending on the filter type, age and condition. We also evaluated point-of-use filter that were in use at 11 out of the 42 households, where tap water was sampled. Bottled water was also extracted and tested for comparison with the tap and filtered water.

## Methods

### Water samples

The sites were selected and sampling was performed as described previously [32]. 42 water samples were collected from taps in homes across different postcodes in the BMA. The sample codes are the postcodes. In the homes of postcodes 08001, 08006, 08008, 08013, and 08017 pitcher-type AC filters and in 08028 faucet AC filters were also sampled to obtain a snapshot of the realistic exposure. Reverse osmosis (RO) was used in the homes sampled in postcodes 08002, 08018, 08019, 08024, and 08029 and both tap water and RO water were sampled. Out of the 10 bottled water samples from the previous work [32], 9 were tested in the bioassays.

### Physical parameters

The pH, total hardness was measured as CaCO_3_ using EDTA titration, free chlorine and total chlorine, conductivity and total organic carbon (TOC) had been reported by Redondo-Hasselerharm et al. [32]. and are reprinted in Table S1.

### Chemical analysis

Concentrations of THMs, HAAs, HANs, HKs and TCNM as well as chlorite and chlorate had been reported by Redondo-Hasselerharm et al. [32]. Inorganic chlorite and chlorate cannot be enriched by SPE. Since THMs and TCNM are too volatile to be captured by SPE and would require specific bioassay formats for volatile chemicals [35], the comparison with bioassay data focused on HAAs, HKs and HANs, and the detected concentrations [32] are reprinted in Table S2. This is also justified because volatile DBPs are often less cytotoxicity than non-volatile DBPs [36] and under real-life scenarios the volatile DBPs contribute less to mixture toxicity [28, 37]. The detected DBPs were MBAA, bromoacetic acid; DCAA, dichloroacetic Acid; BCAA, bromochloroacetic acid; DBAA, dibromoacetic acid; TCAA, trichloroacetic acid; BDCAA, bromodichloroacetic acid; DBCAA, dibromochloroacetic acid; TBAA,tribromoacetic acid; 1,1,1-TCP, 1,1,1-trichloropropanone(acetone); DCAN, dichloroacetonitrile; BCAN, bromochloroacetonitrile; DBAN, dibromoacetonitrile [32].

### Sample preparation for bioassays

Samples were extracted with SPE at the ICRA laboratories. Samples were acidified with HCl to reach pH 2.5–3. Two liters of water were enriched for each sample using 12 cc 500 mg Oasis HLB SPE cartridges. SPE blanks were 2 L of ultrapure water (HPLC grade) run in parallel to the samples in the same setups. The cartridges were dried under vacuum and sent at room temperature to UFZ Leipzig. They were stored at −20 °C prior to elution. The cartridges were eluted without vacuum with 20 mL of ethyl acetate followed by 10 mL methanol, then all extracts were blown down and resolubilized with 1 mL methanol.

### Relative enrichment factor of the samples

All samples were enriched from 2 L to 1 mL, yielding an enrichment factor of the SPE of 2000. An aliquot of the enriched sample extract was then added to a dosing vial, the solvent was blown down to dryness and the sample was resolubilized with cell assay media. Therefore, the bioassay contained no residual solvent. A solvent blank using the same volume of ethyl acetate and methanol (10 mL each), blown down and reconstituted with medium, was also run to ensure that there were no interferences from residuals in the solvents.

The sample was transferred from the dosing vial into a 96 well plate and serially diluted in test media and 30 µL of this dosing solution was transferred to 384-well plates that contain cells in 10 µL medium.

The final relative enrichment factor (REF) is the combination of the enrichment of the extract and the dilution in the bioassay [38] and represents the enrichment of the original water sample in each bioassay. The REF is equivalent to concentration and is expressed in the units [L_water sample_/L_bioassay_].

### AREc32 assay for activation of oxidative stress response

The SPE extracts were tested in the AREc32 assay for activation of oxidative stress response. The AREc32 assay was performed according to [39] with some modifications. Briefly, the extracts were serially diluted in DMEM with 10% fetal bovine serum (FBS) and added to a 384 well plate containing cells at a final density of 8.33 × 10^4^ cells/mL. The plates were incubated at 37 °C for 24 h, then luciferase production was measured using luciferin and ATP as substrate and luminescence relative light units RLU were recorded. The measure of effect was the induction ratio IR, which is ratio of the RLU of the sample at a given concentration divided by the mean of the RLU of the unexposed cells.

Live-cell analysis using IncuCyte S3 live cell imaging system (Essen BioScience, Ann Arbor, Michigan, USA) was used to assess confluency, which served as a proxy for cytotoxicity. Confluency was measured 48 h after seeding (24 h after dosing) using phase contrast images [40]. The extracts were tested up to a REF of 500. tert-Butylhydroquinone (tBHQ) was the positive reference compound for AREc32.

An inhibitory concentration IC_10_ for 10% cytotoxicity was derived from the confluency measurements using a linear concentration-response regression with intercept of 0 according to [40]. The cytotoxicity can also be expressed as toxic units TU_bio_, which is the inverse of the IC_10_.

$${{{{{{{\mathrm{TU}}}}}}}}_{{{{{{{{\mathrm{bio}}}}}}}}} = \frac{1}{{{{{{{{{\mathrm{IC}}}}}}}}_{{{{{{{{\mathrm{10}}}}}}}}}}}$$

Only concentrations below IC_10_ and up to a maximum IR of 5 were used for concentration-response assessment of the activation of ARE. Here we applied a linear concentration-response regression with intercept of IR 1 [39]. An IR of 1.5 corresponds to a 50% increase over the IR of the unexposed cells, and the associated concentration was used as effect concentration EC_IR1.5_ [39].

### Neurite outgrowth inhibition assay

The SPE extracts were tested in a neurotoxicity assay that was based on the cytotoxicity and neurite outgrowth inhibition in differentiated SH-SY5Y cells obtained from Sigma-Aldrich, 94030304 [27]. Briefly, the cells were plated in a collagen-coated black/clear flat bottom 384-well plate (Corning, 354667) and exposed with the serially diluted extracts. Prior to testing the methanol extracts were solvent-exchanged into medium to assure that the effects were not impacted by the presence of solvents. The extracts were tested in 11 different concentrations up to a REF of 300. After incubation of the plate at 37 °C for 24 h, image analysis was performed with IncuCyte S3. Cell viability was quantified from fluorescence images after staining with Nuclear Green LCS1 (Abcam, ab138904) and propidium iodide (Sigma-Aldrich, 81845). The number of total and dead cells were derived to determine cytotoxicity in neuronal cells.

Inhibition in neurite outgrowth was measured using phase-contrast image and the length of neurite was quantified with IncuCyte NeuroTrack software module. 10% effect concentration for cytotoxicity and neurite outgrowth inhibition were expressed as IC_10_ and EC_10_, respectively. Narciclasine was used as positive reference compound for neurotoxicity assay.

### Direct testing of unenriched water samples in the neurite outgrowth inhibition assay

The water was also tested in its entirety after filtration in another setup of the neurite outgrowth inhibition in differentiated SH-SY5Y cells [27]. The experimental procedure is detailed in Text S1.

### Iceberg modelling for oxidative stress response

Bioanalytical equivalent concentrations (BEQ) can be used to compare the predicted biological effect from the concentrations of the detected DBPs with the biological effect. The concept of BEQ implies that chemicals act concentration-additive as was demonstrated multiple times for designed complex mixtures in equipotent concentration ratios and concentration ratios of occurrence for the AREc32 assay [37, 41, 42]. The BEQ_bio_ can be derived directly from the effect concentration measured in the sample by relating it to the effect concentration of a reference chemical [38]. In previous work we have used MCAA as reference compound for DBPs [43] but given that MCAA was below the limit of detection in all samples and it is of low potency we used DBAN, the most potent DPB in the AREc32 assay among the tested DBPs [19] as reference chemical, as was also done in a previous mixture study [37]. The DBAN-EQ_bio_ can then be calculated by Eq. 2 with EC_IR1.5_ of 0.15 µM or 29 µg/L of DBAN.

1 $${{{{{{{\mathrm{DBAN}}}}}}}}-{{{{{{{\mathrm{EQ}}}}}}}}_{{{{{{{{\mathrm{bio}}}}}}}}} = \frac{{{{{{{{{\mathrm{EC}}}}}}}}_{{{{{{{{\mathrm{IR1}}}}}}}}{{{{{{{\mathrm{.5}}}}}}}}}{{{{{{{\mathrm{(DBAN)}}}}}}}}}}{{{{{{{{{\mathrm{EC}}}}}}}}_{{{{{{{{\mathrm{IR1}}}}}}}}{{{{{{{\mathrm{.5}}}}}}}}}({{{{{{{\mathrm{water}}}}}}}}\,{{{{{{{\mathrm{extract}}}}}}}})}}$$

If the concentration of the detected DBPs *i*, *C*_*i*_, is multiplied by their relative effect potency REP_*i*_, we obtain the DBAN equivalent concentration of this DBP *i*, DBAN-EQ_chem_(*i*). If all DBPs act concentration-additive in mixtures, which has been established for drinking water DBPs in the AREc32 assay [37], the DBAN-EQ_chem_(*i*) can be summed up to yield the measure of the mixture effect of the detected DBPs, DBAN-EQ_chem._(Eq. 2).

2 $${{{{{{{\mathrm{DBAN}}}}}}}} - {{{{{{{\mathrm{EQ}}}}}}}}_{{{{{{{{\mathrm{chem}}}}}}}}}	 = \mathop {\sum}\limits_{i = 1}^n {{{{{{{{\mathrm{DBAN}}}}}}}} - {{{{{{{\mathrm{EQ}}}}}}}}_{{{{{{{{\mathrm{chem}}}}}}}}}} \left( i \right) \\ 	 = \mathop {\sum}\limits_{i = 1}^n {\frac{{{{{{{{{\mathrm{EC}}}}}}}}_{{{{{{{{\mathrm{IR}}}}}}}}1.5}({{{{{{{\mathrm{DBAN}}}}}}}})}}{{{{{{{{{\mathrm{EC}}}}}}}}_{{{{{{{{\mathrm{IR}}}}}}}}1.5}(i)}}} \cdot {{{{{{{\mathrm{C}}}}}}}}_i = \mathop {\sum}\limits_{i = 1}^n {{{{{{{{\mathrm{REP}}}}}}}}_i \cdot C_i}$$

The mixture effect from the detected chemicals DBAN-EQ_chem_ can now be compared with the measured mixture effect in the bioassay expressed as DBAN-EQ_bio_ to assess the contribution of the detected chemicals (Eq. 3).

3 $$\% \,{{{{{{{\mathrm{effect}}}}}}}}\,{{{{{{{\mathrm{explained}}}}}}}}\,{{{{{{{\mathrm{by}}}}}}}}\,{{{{{{{\mathrm{detected}}}}}}}}\,{{{{{{{\mathrm{chemicals}}}}}}}} = \frac{{{{{{{{{\mathrm{DBAN}}}}}}}} - {{{{{{{\mathrm{EQ}}}}}}}}_{{{{{{{{\mathrm{chem}}}}}}}}}}}{{{{{{{{{\mathrm{DBAN}}}}}}}} - {{{{{{{\mathrm{EQ}}}}}}}}_{{{{{{{{\mathrm{bio}}}}}}}}}}}$$

## Results

### Oxidative stress response

Some of the extracts were slightly acidic, as evidenced by a discoloration of the phenol red medium and the pH of the bioassay medium had to be neutralized with 0.3 to 0.5 µL of 5 M NaOH solution. The pH shift could only be measured with pH paper because the sample volume of 120 µL of the dosing vial was too low to use a pH electrode.

The acidity in the highly enriched samples might have well been caused by the dissociation of the HAAs when dissolving the neutral form of the HAA eluted from the SPE cartridge with solvent in medium that is only weakly buffered at pH 7.4, which would lead to a dissociation of the HAA that leads to a proton release and hence acidification of the medium. In the dosing vial, the sample had an REF of 2000 and the decrease in pH was higher for samples with high sum concentrations of HAAs (Fig. S1). The samples with the highest acidification had sum concentrations of HAAs of 0.05 to 0.25 µM at REF 1, which corresponds to 100 to 500 µM HAA at REF 2000, which could bring about such a pH shift, when the HAAs deprotonate in the medium that is weakly buffered at pH 7.4.

Although all highly enriched SPE extracts of the water samples were slightly acidic, the acidity of the extracts was not caused by the experimental procedure because blanks, bottled water and water filtered by RO did not exhibit pH shifts, while the original tap water and the AC-filtered water had pH values lowered by one to two pH units (Fig. S1).

Even if the pH were not optimal, the AREc32 assay results would not be impacted as we have demonstrated that AREc32 can be adapted to growth on medium from pH 6.5 to pH 8 without change in sensitivity towards chemicals and cells that were not adapted but challenged with pH during testing also delivered robust results (unpublished data). This is different from the neurotoxicity assay, which is sensitive to pH shifts as described below.

The positive control *tert*-butylhydroquinone (tBHQ) in the AREc32 assay had an EC_IR1.5_ of 8.34 ± 0.15 µM (Fig. S2a, Table S3), which is in the range of previous literature data [39]. SPE blanks had EC_IR1.5_ values ranging from REF 81 to > 500 (Fig. S2b, Table S3), i.e., were much less potent than the tap water, but in the same range of effects of RO water and bottled water. The solvent blanks showed no effects (Fig. S2c, Table S3).

**Fig. 1:**
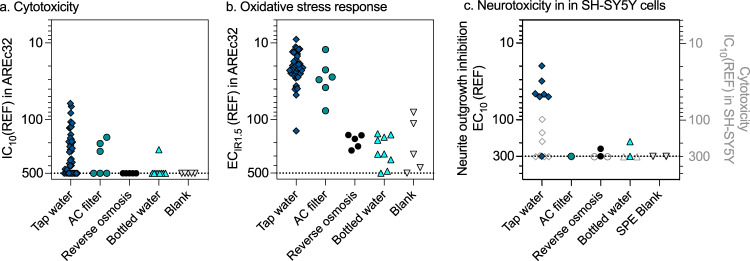
Cytotoxicity, oxidative stress, and neurotoxicity. Comparison of the effect concentrations of the different water types: **a** IC_10_ for cytotoxicity in AREc32. **b** EC_IR1.5_ for oxidative stress response in AREc32. **c** EC_10_ for neurite outgrowth inhibition in SH-SY5Y cells (left y-axis) and IC_10_ for cytotoxicity in SH-SY5Y cells (right y-axis, grey symbols). Data are in Tables S1, S2. The line is at REF 500/300, the highest tested concentration in AREc32/SH-SY5Y and the symbols stand for experiments without detected effect ( > 500/ > 300).

None of the samples showed cytotoxicity (Fig. 1a) or activation of oxidative stress response (Fig. 1b) without substantial enrichment. The tap water had EC_IR1.5_ ranging from REF 9 to 141 (example of one concentration-response curve in Fig. S2d, EC_IR1.5_ in Table S3). Cytotoxicity occurred at 3 to 34 times higher REF (IC_10_ for cytotoxicity in Table S3) than activation of oxidative stress response. 12 of 42 tap water samples were not cytotoxic up to REF 500, which means that the tap water showed a rather specific oxidative stress response. Water treated with AC filters had similar oxidative stress response before and after filtration and cytotoxicity was small and not impacted by filtration (Fig. 1a, b, Table S3). As discussed by Redondo-Hasselerharm et al. [32], these were pitcher-type AC filters that were in households, so it cannot be assured that they were well maintained and not overloaded.

After reverse osmosis even the low cytotoxicity had disappeared (IC_10_ > 500) and oxidative stress response was active only at REF > 150 (Fig. 1, Table S3). Bottled water showed no cytotoxicity up to REF 500 with exception of one out of 9 bottles, with an IC_10_ of 246 and activation of oxidative stress response had EC_IR1.5_ > REF 100 (example of one concentration-response curve in Fig. S2e, Fig. 1, Table S3).

Upon direct comparison of sample types (Fig. 1b), it is evident, that tap water and tap water after AC filtration had the same ranges of effect concentrations and the water had to enriched 10 to 100-fold to case the 10% activation of the oxidative stress response pathway (exception 08021, which had an EC_IR1.5_ > 100). Reverse osmosis-treated tap water and bottled water had consistently lower effects in the range of the SPE blanks. One of five SPE blanks had a slightly increased effect, the source of which could not be identified.

**Fig. 2:**
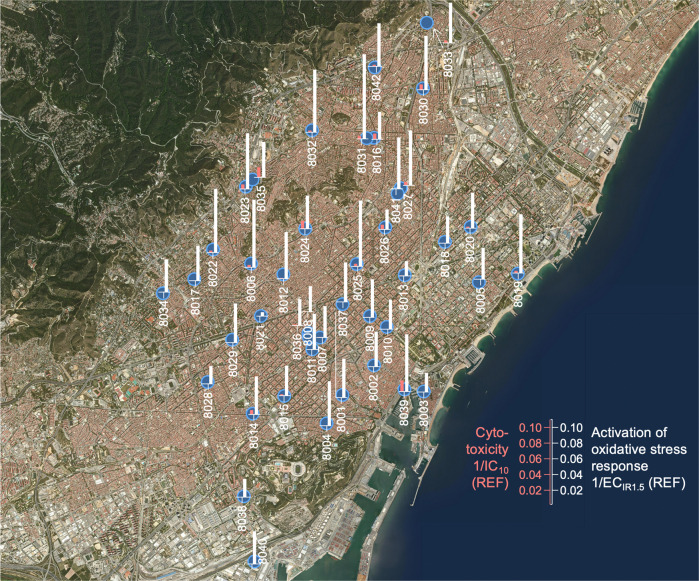
Mixture effects. Distribution of mixture effects across the drinking water supply of Barcelona Metropolitan Area (BMA) expressed as inhibitory concentration IC_10_ for 10% reduction of cell viability in the AREc32 cell line and effect concentration EC_IR1.5_ for activation of oxidative stress response. Data are from Table S3. The units are relative enrichment factors REF, and samples that did not show cytotoxicity up to a 500-fold enrichment had no bar. The y-axis is inverse (1/IC_10_ or 1/EC_IR1.5_) because a low IC_10_/EC_IR1.5_ refers to a high cytotoxicity or effect but the legend refers to the original IC_10_/EC_IR1.5_.

Fig. 2 shows the spatial distribution of cytotoxicity and oxidative stress response on a map of the BMA. On first sight, the distribution of effects appears quite uniform across the entire BMA with slightly higher activation of oxidative stress response in the Northwestern region of BMA, where also some cytotoxicity was detectable. The site 08039 was higher than other sites at the coastal region but that was a public water fountain where the highest concentration of DBAA (13.9 µg/L) and TBAA (6.6 µg/L) and DBAN (4.2 µg/L) were measured. Bromine containing DBPs are known to be more toxic than their chlorinated analogues [44] and, in particular, DBAN is the most cytotoxic DBP with the highest activation of oxidative stress response included in the study (Table S5 [19]).

### Neurotoxicity

#### Tap water samples

None of the directly tested tap water samples showed any neurotoxic effects (Text S2). Unenriched water did not induce diminution of cell viability (MTT) or increase of LDH leakage (Fig. S3), nor did they increase the propidium iodide permeabilization (Fig. S4). The neuronal phenotype differentiation and neurite length of the differentiated SH-SY5Y cells were not affected.

#### SPE extracts

Only selected SPE extracts were evaluated with the screening neurotoxicity assay that was tuned for water quality assessment [45] because of the challenge of the acidity of the SPE extracts described below. The concentration-response curves for cytotoxicity and inhibition of neurite outgrowth are depicted in Fig. S5.

The EC_10_ for neurite outgrowth inhibition of the positive control narciclasine was 9 nM (Table S4), which is consistent with earlier work. The SPE blanks showed no cytotoxicity nor effects up to REF 300. The high acidity of some of the extracts posed a problem for this assay despite the attempt to neutralize the sample after dissolving in medium and before dosing with cell debris as is visible in the images of the neutralized extract of tap water 08008, while tap water 08022 only showed decreased neurite numbers and lengths and decreased cell number as compared to the unexposed cells (Fig. S6).

Apart from 08025, which had an IC_10_ of 98 (Table S4), none of the tested samples caused cytotoxicity up to a REF of 100, three tap water samples and all filtered and bottled water even up to a REF of 300. The neurite outgrowth was inhibited by all tap water samples (with exception of 08008, which had shown the pH problems) and by none of the other samples. The EC_10_ for neurite growth inhibition ranged from 20 to 51, which means the water had to be enriched 20 to 51 times to show 10% neurotoxic effects.

Although fewer samples were tested in the neurotoxicity assays due to the pH shift issue, the same picture emerged (Fig. 1c) as for AREc32 (Fig. 1a, b), although the AC filters removed the neuroactive chemicals to below the limit of detection. We have not fingerprinted single DPBs in this neurotoxicity assay yet, but it is often highly responsive to pesticides and other water pollutants that are more hydrophobic [45], so that the sorption to AC might have been more efficient for the chemicals causing neurotoxicity.

## Discussion

### Comparison of oxidative stress response with literature data and other water types

The oxidative stress response was in the same range or slightly higher than in previously tested drinking water (Hebert et al. [46] and Neale et al. [10]) but clearly less active than in surface water during rain events [45] and wastewater treatment plant effluent (WWTP) (Fig. S7a). None of the tap water samples exceeded the proposed effect-based trigger value EBT-EC_IR1.5_ of 6 (REF) [41].

AREc32 can be impacted by both, DBPs and micropollutants, but most of the oxidative stress response in unchlorinated samples remained unexplained to date [41]. If one measures activation of the oxidative stress response directly before and after chlorination, it is possible to derive the contribution of DBPs to the overall effect, but this is not feasible here because we only sampled the treated or treated and filtered tap water, which is much more realistic of human exposure than previous work on drinking water treatment plants and assessment of DBP formation potential [47].

There was no direct correlation between cytotoxicity and activation of oxidative stress response (Fig. S8a). Cytotoxicity can be considered an effect-scaled sum parameter for all chemicals present in a sample and acting together, while only a fraction of micropollutants [48] and many but not all DBPs [19] activate the oxidative stress response.

Organic matter plays an important role for the formation of DBPs [47] but neither the cytotoxicity (Fig. S8b) nor the activation of the oxidative stress response (Fig. S8c) was directly correlated with the TOC.

### Neurotoxicity

To our knowledge no neurotoxicity assay has been applied to drinking water samples yet, so we can only compare to other water types. The tap water showed equal to lower effects compared to wastewater treatment plant effluent and surface water collected during rain events (Fig. S7b). As effect concentrations for individual DBPs in the neurotoxicity assay are not available, iceberg modelling could not be performed. Only one extract of water from postcode 08008 was tested in the neurotoxicity assay before and after AC filtration (Table S4) but due to the acidity only cell debris were observed. In contrast, after RO no effects could be observed (Table S4) and there were no issues with acidification, but no matching tap water sample could be tested due to limited sample volume availability.

### Comparison of detected DBPs and oxidative stress response

Iceberg modelling helps to understand the contribution of individual detected DBPs to the measured effect and how much of the mixture effect is contributed to by DBPs and micropollutants not quantified or not toxicologically characterized. Here we performed the iceberg modelling with the detected concentrations of HAAs, HKs and HANs (Table S2 [32]), and their EC_IR1.5_ were taken from the literature (Table S5 [19]).

**Fig. 3:**
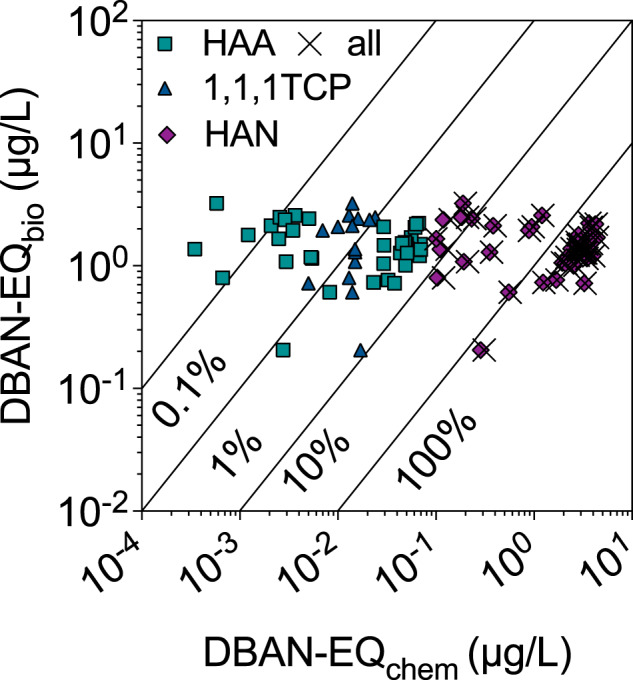
Iceberg modeling. Comparison of the bioanalytical equivalent concentrations DBAN-EQ_bio_ with the DBAN-EQ_chem_ and percentage of bioassay response explained by the detected chemicals. All data in Table S6.

The DBAN-EQ_chem_ reported in Table S6 comprise the contribution of HAAs, HKs and HANs to the mixture effect. The sum of the DBAN-EQ_chem_(*i*) of the HAAs, DBAN-EQ_chem_(ΣHAA), contributed very little to the DBAN-EQ_chem_ (Eq. 3, Fig. 3). DBAN-EQ_chem_ was dominated by HANs (DBAN-EQ_chem_(ΣHAN)), and particularly the most potent DBAN (Fig. 3).

DBAN-EQ_chem_(ΣHAN) explained 5% to 460% of the DBAN-EQ_bio_, while the HAA explained a mere 0.005% to 5.6% of the DBAN-EQ_bio_. If present at all, the HK 1,1,1TCP contributed 8.4% to the DBAN-EQ_bio_ at postcode 08020 but was typically < 1% (Fig. 3).

That DBAN-EQ_chem_ exceeded DBAN-EQ_bio_ by up to a factor of 4 (Table S6) is most likely caused by differences in sample preparation methods. The DBPs were extracted with group-specific sample preparation and targeted analytical methods [32], while the water was extracted with SPE. HANs are a technically challenging group of DBPs: HANs are not volatile enough to be captured by a purge and trap method but are also not well recovered by SPE [28]. For chemical analysis HANs were extracted by liquid-liquid salted microextraction and gas chromatography but this extraction method would not keep the mixture intact and not extract HAAs. Therefore, for water sample preparation for bioanalysis, SPE remains the preferred method. Even if it does not capture all individual DBPs with high yield, it captures a bigger diversity than any specialized extraction methods [28]. The SPE recovery of DCAN was negligible and that of DBAN around 20% [28], which would explain the factor 4 overestimation of DBAN-EQ_chem_. HAA were recovered by SPE between 10 and 66% [28]. In addition, HANs are instable in bioassay medium and form HAAs [49]. Mono-HANs are fairly stable, but Di-HANs have been reported to be reduced by 40% (DBAN), 68% (BCAN) and 85% (DCAN) in plate-based bioassays and cell culture medium with 10% fetal bovine serum [49]. As the HAAs are less potent than the corresponding HANs, this would also explain part of the overestimation of DBAN-EQ_bio_ by DBAN-EQ_chem_.

The THMs chloroform (TCM), bromodichloromethane (BDCM), dibromochloromethane (DBCM) and bromoform (TBM) had also been included in the analysis in the previous study [32]. Due to their high volatility, they were not extracted by SPE [28], but one can estimate their additional effect load. Despite the relatively high concentrations of 0.31 to 36.3 µg/L for TCM, 0.32 to 11.5 µg/L for BDCM, 1.7 to 26.5 µg/L for DBCM and 0.17 to 57.7 µg/L for TBM [32], due to the low effect potencies [19], the sum of THMs would add less than 1% (0.04 to 0.87%) additional DBAN-EQ_chem_ to the DBAN-EQ_bio_ of the extracted mixtures, which is negligible (data and calculations not shown). This is consistent with what has been found in previous work where THMs were negligible contributors to mixture effects [28, 37, 46].

Cytotoxicity was 3 to 23 times less sensitive than the activation of oxidative stress response and therefore many samples were not active up to REF 500. A comprehensive study of drinking water from 6 US drinking water treatment plants found that the mammalian cell cytotoxicity index (CTI), which is equivalent to the TU used in the present study, was also dominated by the HANs [50].

### Spatial distribution of DBPs and effects

BMA receives water from Ter and Llobregat river, the latter being richer in bromide concentration [51]. Therefore, the relative portion of Br-BPs vs Cl-DBPs varies greatly within the region studied as reported by Redondo-Hasselerharm et al. [32]. In the Northwestern area of BMA, the water comes from the Ter river with a lower concentration of bromide. There was a clear spatial distribution of concentrations with chlorinated DBPs occurring at higher concentrations in the North, brominated DBPs rather in the South and East and 1,1,1TPP only in the Northwest (Fig. S9). While HAAs and the HK were not detected in several sampling sites, HANs were ubiquitous (Fig. S9). The concentrations in Fig. S9 were already converted to DBAN-EQ_chem_(*i*) to allow a direct comparison between effect contribution of individual DBPs.

In the Northwestern area of BMA (postcodes 08006, 08013, 08016, 08021 to 08027, 08031, 08032, 08035 and 08041, 08042) concentrations of DCAA, BCAA, DBAA, TCAA were substantially higher than that of all HANs (ratio ΣHAA/ΣHAN > 9) but due to the higher REP of HANs, the DBAN-EQ_chem_(ΣHAN) were 32 to 5388 times higher than DBAN-EQ_chem_(ΣHAA). In these samples the concentration ratios DBAN/DCAN were smaller than 1, often even smaller than 0.1 or no DBAN detected.

**Fig. 4:**
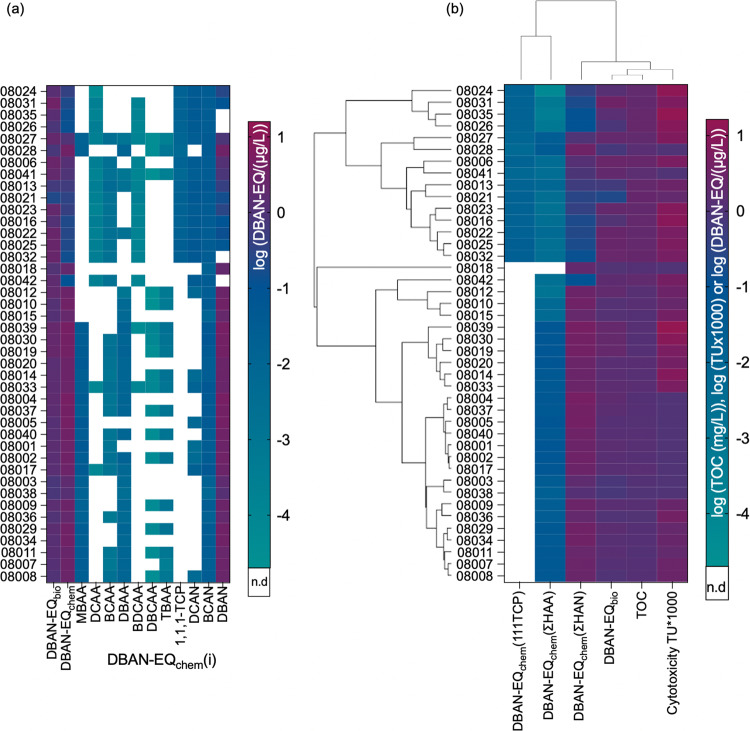
Iceberg modeling and sample clustering. **a** Iceberg modeling: comparison of the bioanalytical equivalent concentrations DBAN-EQ_bio_ with the DBAN-EQ_chem_ (Table S6), and the contribution of the individual quantified DBPs *i*, DBAN-EQ_bio_(*i*). All concentrations are from Table S2, effect data of the single chemicals from Table S5. MCAA was not detected, TCAA was not active in the bioassay, and they were therefore omitted in the plot. The clustering was adjusted to the hierarchical clustering of **b**. **b** Hierarchical clustering of measured effects expressed as DBAN-EQ_bio_, predicted effects DBAN-EQ_chem_(ΣHAA), DBAN-EQ_chem_(ΣHAN), DBAN-EQ_chem_(1,1,1-TCP), cytotoxicity toxic units TU (scaled by 1000 for better visualization) and total organic carbon (TOC).

Despite these regional difference in contribution of Cl-DBPs and Br-DBPs, the DBAN-EQ_chem_ was always dominated by HANs due to their high potency, and HAAs had only a minor contribution to DBAN-EQ_chem_ (Fig. 4a and Table S6). 1,1,1TCP was only detected in a few samples that were at the same time very low in HAA in the postcodes 08006, 08013, 08016, 08021 to 08028, 08031, 08032, 080315, 08041. Sample 08018 had only HANs and no detected HAAs or 1,1,1-TCP but was as potent as other samples. In the coastal areas (postcodes 08001 to 08005, 08007 to 08011, 08014, 08018 to 08020, 08039) DBAN was 10 times higher than DCAN or no DCAN was detected and HAAs were in a similar concentration range as HANs.

Hierarchical clustering depicted in Fig. 4a only clustered the sample locations and depicts DBAN-EQ_bio_, DBAN-EQ_chem_ and DBAN-EQ_chem_(*i*). In the Northwestern area of BMA (Fig. 2, postcodes 08006, 08016, 08022 to 08026, 08031, 08032, 08035 and 08041, 08042) less than 50% of the effect could be explained by the HANs presumably due to the absence or low concentration of the brominated DBAN, but the HAAs also had less than 0.1% contribution to the mixture effects (Fig. 4a). However, 1,1,1-TCP was detected at these sites and contributed 0.4 to 8.4% of the biological effect. Presumably other DBPs that were not in the target analysis or not characterized in the AREc32 could have contributed to the mixture effect.

In Fig. 4b, the DBAN-EQ_chem_ were compared with DBAN-EQ_bio_ as well as TOC and cytotoxicity DBAN-EQ_chem_(ΣHAN). The original clustering was performed after scaling (Fig. S10) but in Fig. 4b, the original data are plotted on a logarithmic scale for easier comparison. DBAN-EQ_chem_(1,1,1-TCP) and DBAN-EQ_chem_(ΣHAA) clustered together and were an independent group from all others, indicating that they did not influence the mixture effect very much due to their low potency. DBAN-EQ_bio_ then clustered with the TOC concentration, which can be explained by TOC being an important precursor of DBPs, although we had not observed a correlation between TOC and DBAN-EQ_bio_. Those two were associated with cytotoxicity on the next level of clustering, while the cluster with DBAN-EQ_chem_(ΣHAN) connected all of them on the highest level.

### Efficacy of point-of-use filters

Consistent with the observations in the cell assays, where the AC filters did not reduce the cytotoxicity and effects, the filtration also had a low and variable effect on the removal of TOC and DBPs. Three of six AC filter did not change the TOC concentrations, only two lowered it and one even increased the TOC by 38% (Table S7). While the concentrations of HAAs were reduced by 18 to 91% and those of HANs by 47 to 100%, this did not directly translate to the reduction of the predicted mixture effect DBAN-EQ_chem_ because different DBPs were detected before and after the filter and there was no consistent picture on the removal of DBAN-EQ_chem_. Sometimes the more potent DBPs were removed, sometimes those of lower potency.

What is striking is that the measured mixture effect for oxidative stress response (DBAN-EQ_bio_) was only decreased by 49 to 72% in three filters, but one filter showed no removal (4%) and for one filter the toxicity increased by 44% and for another one it tripled. As these filters were not run under optimal conditions but reflect real-life scenarios, we can conclude as in the previous study [32] that domestic filters need to be used according to the manufacturer’s instruction and sufficiently often replaced when the AC become saturated and DBPs and other chemicals can break through. The filter that showed a three-times increased oxidative stress response had a recent cartridge change reported by the participant (personal communication).

In contrast, the five RO filters all reduced the TOC (Table S1) substantially and no more DBPs were detected (Table S2). This aligns well with the substantial but not full reduction of mixture effects described by DBAN-EQ_bio_, which were reduced by 88 to 92% (Table S7).

## Significance

This study builds up on three earlier communications of the presence of DBPs [32], micropollutants [33] and micro(nano) plastic [34] in the same water samples but provides important additional insights into the potential harm DBPs may cause. Bioassays are useful screening tools for drinking water quality and removal of DBPs. Even if DBPs fall below the limit of detection, their concentrations are likely not zero but the non-detectable DBPs still contribute to the mixture effect. Moreover, there are many more DBPs than typically covered by chemical analysis and we do not even know how many there really are. Therefore, bioassays are complementary to chemical analysis and can be used to assess the overall burden of DBPs already scaled for effect-potency.

Although the pattern, type and concentration of DBPs showed a substantial regional variability, the effects were much less variable across the entire BMA. The mixture effects were mainly dominated by non-regulated DBPs.

We propose to complement the chemical analysis of regulated DBPs by bioassays because they provide a sum parameter for all DBPs and other micropollutants present in a drinking water sample. This requires the definition of effect-based trigger (EBT) values, which differentiate acceptable from poor water quality. Tentative EBTs values for oxidative stress response already exist and none of the investigated water samples exceeded these EBTs. Before bioassays can be used for regulatory drinking water monitoring EBTs must be defined for all bioassays of relevance for DBPs.

### Supplementary information

Supplementary Information

## Data Availability

The data generated and analyzed during this study can be found in within the published article, reference [32] and in the Supplementary Information file.
