# Supplementary material for: In vitro bioassays for monitoring drinking water quality of tap water, domestic filtration and bottled water
Source: J Expo Sci Environ Epidemiol. 2023 Jun 16;34(1):126–35. doi: 10.1038/s41370-023-00566-6 (PMC10907286; doi:10.1038/s41370-023-00566-6)
Supplement: Supplementary file 1 — Supplementary Information [file 41370_2023_566_MOESM1_ESM.pdf]

Supplementary Information

In vitro bioassays for monitoring drinking water quality of tap water, domestic filtration and bottled water

Beate I. Escher[1,2], Jordi Blanco[3], Josep Caixach[4], Dora Cserbik[5,6,7], Maria J. Farré[8,9], Cintia Flores[4], Maria König[1], Jungeun Lee[1], Jo Nyffeler[1], Carles Planas[4], Paula E. Redondo-Hasselerharm[5,6,7], Joaquim Rovira[3,10], Josep Sanchís[8,9#], Marta Schuhmacher[10], Cristina M. Villanueva [5,6,7,11]

[1]Helmholtz Centre for Environmental Research – UFZ, Department of Cell Toxicology, Leipzig, Germany

[2]Eberhard Karls University Tübingen, Environmental Toxicology, Department of Geosciences, Tübingen, Germany

[3]Laboratory of Toxicology and Environmental Health, School of Medicine, Universitat Rovira i Virgili, Reus, Spain

[4]Mass Spectrometry Laboratory/Organic Pollutants, Institute of Environmental Assessment and Water Research, IDAEA-CSIC, Barcelona, Spain

[5]ISGlobal, Barcelona, Spain

[6]Universitat Pompeu Fabra, UPF, Barcelona, Spain

[7]CIBER Epidemiología y Salud Pública, CIBERESP, Madrid, Spain

[8]Catalan Institute for Water Research, ICRA, Girona, Spain

[9]University of Girona, Girona, Spain

[10]Environmental Engineering Laboratory, Universitat Rovira i Virgili, Tarragona, Spain

[11]Hospital del Mar Medical Research Institute, IMIM, Barcelona, Spain

Table of Content (in order of occurrence)

Table S1. Physical parameters of the water (data reprinted from Redondo-Hasselerharm et al. [1].

Table S2. Analytical results (data reprinted from Redondo-Hasselerharm et al. [1]).

Text S1. Additional information on the experimental method for direct neurotoxicity measurement with unenriched water.

Figure S1. pH shift towards lower pH appears to be related to the sum concentrations of haloacetic acids (HAA).

Figure S2. Concentration-response curves in AREc32 (selected examples).

Table S3. Inhibitory concentration IC_{10} for 10 % reduction of cell viability in the AREc32 cell line and effect concentration $EC_{IR1.5}$ for activation of oxidative stress response of the water samples.

Text S2. Results of the direct neurotoxicity measurement with unenriched water.

Figure S3. Cytotoxicity of SH-SY5Y dosed directly with water.

Figure S4. SH-SY5Y cells neurite outgrowth inhibition assessment with directly dosed water.

Figure S5. Concentration-response curves in the neurotoxicity assay dosed with enriched water extracts.

Figure S6. Effect of acidity of some of the samples in the neurotoxicity assay.

Table S4. Inhibitory concentration IC_{10} for 10 % reduction of cell viability in the neuronal cell line SH-SY5Y and effect concentration EC_{10} for reduction of neurite length by 10% for selected samples.

Figure S7. Comparison of the effect concentrations of tap water effect data of this study with literature of drinking water and other water types.

Figure S8. Comparison of cytotoxicity, oxidative stress response and total organic carbon (TOC) of the tap water.

Table S5. Effect concentrations $EC_{IR1.5}$ and cytotoxicity IC_{10} for the detected chemicals in AREc32.

Table S6. Iceberg modeling.

Figure S9. Spatial distribution of the DBAN-$EQ_{chem}(i)$ of (a) MBAA, (b) DCAA, (c) BCAA, (d) DBAA, (e) BDCAA, (f) DBCAA, (g) TBAA, (h) 1,1,1TCP, (i) DCAN, (j) BCAN, (k) DBAN and (l) the sum of DBAN-$EQ_{chem}(i)$, DBAN-EQ_{chem}.

Figure S10. Hierarchical clustering after scaling of measured effects.

Table S7. Percent reduction of total organic carbon (TOC), sum of concentrations of haloacetic acids (HAA) and haloacetonitriles (HAN) and reduction of predicted mixture effects.

Table S1. Physical parameters of the water including pH, hardness (mg $CaCO_3$/L) , free chlorine (mg/L), total chlorine (mg/L), conductivity (μS/cm) and total organic carbon (TOC, mg/L). Data reprinted from Redondo-Hasselerharm et al. [1].

Tap water (postcode)	pH	Hardness	Free chlorine	Total chlorine	Conductivity	TOC
08001	7.75	255	n.a.	n.a.	937	1.3
08002	7.61	270	1.4	1.9	951	1.2
08003	7.75	300	n.a.	n.a.	1121	1.1
08004	7.27	310	1.2	1.9	981	1.2
08005	7.75	300	n.a.	n.a.	939	1.2
08006	8.00	168	n.a.	n.a.	417	2.3
08007	6.96	300	1.2	1.8	957	1.2
08008	7.33	255	2.4	2.7	914	1.2
08009	7.26	243	1.1	1.7	840	1.3
08010	7.51	300	1.3	2.3	838	1.3
08011	7.61	240	1.8	2.3	788	1.1
08012	7.80	285	n.a.	n.a.	916	1.2
08013	7.65	174	n.a.	n.a.	366	2.1
08014	7.48	261	2.3	2.6	907	1.3
08015	7.65	300	n.a.	n.a.	1020	1.2
08016	7.85	165	n.a.	n.a.	368	2.1
08017	7.28	210	1.8	2.1	627	1.4
08018	7.55	300	n.a.	n.a.	984	1.2
08019	7.56	258	1.3	2.1	823	1.2
08020	6.99	246	1.0	2.0	876	1.4
08021	7.90	180	n.a.	n.a.	361	2.3
08022	7.64	180	1.7	2.4	358	1.9
08023	7.64	168	2.1	2.5	353	1.9
08024	7.76	165	<0.02	0.1	364	1.9
08025	7.70	180	n.a.	n.a.	376	2.1
08026	7.95	150	n.a.	n.a.	371	2.3
08027	7.95	150	n.a.	n.a.	374	1.8
08028	7.57	252	1.6	2.5	843	1.4

08029	7.49	288	1.7	2.4	941	1.2
08030	6.24	135	1.8	2.0	591	1.2
08031	6.78	171	0.9	1.7	356	1.9
08032	7.85	178	n.a.	n.a.	379	2.2
08033	6.74	174	2.2	2.4	520	1.4
08034	7.44	285	2.0	2.4	948	1.2
08035	7.90	165	n.a.	n.a.	368	2.2
08036	7.20	300	n.a.	n.a.	1078	1.1
08037	7.45	261	1.9	2.6	828	1.2
08038	7.90	300	n.a.	n.a.	1185	1.0
08039	7.52	291	0.8	1.5	892	1.3
08040	7.53	267	1.1	1.7	1018	1.3
08041	7.80	120	n.a.	n.a.	372	1.5
08042	7.73	156	1.6	2.2	359	1.8
**Activated carbon filters**	**pH**	**Hardness**	**Free_Cl**	**Total_Cl**	**Conductivity**	**TOC**
08001	6.80	105	n.a.	n.a.	673	1.3
08006	6.80	45	n.a.	n.a.	374	1.2
08008	7.40	240	0.2	0.4	872	1.2
08013	6.80	30	n.a.	n.a.	247	2.1
08017	7.26	201	<0.02	0.1	670	1.9
08028	7.45	246	0.1	0.2	838	1.1
**Reverse Osmosis**	**pH**	**Hardness**	**Free_Cl**	**Total_Cl**	**Conductivity**	**TOC**
08002	7.38	30	<0.02	<0.02	64	0.5
08018	6.80	18	n.a.	n.a.	95	0.3
08019	7.70	18	<0.02	<0.02	61	0.2
08024	7.82	5	<0.02	<0.02	16	0.4
08029	7.52	33	<0.02	<0.02	101	0.3
**Bottled water**	**pH**	**Hardness**	**Free_Cl**	**Total_Cl**	**Conductivity**	**TOC**
A	7.17	105	<0.02	<0.02	198	0.4
B	7.29	33.0	<0.02	<0.02	32.0	0.3
C	7.32	60.0	<0.02	<0.02	125	0.4
D	7.00	141	<0.02	<0.02	262	0.4

E	7.30	108	<0.02	<0.02	184	0.2
F	7.67	225	<0.02	<0.02	338	0.4
G	7.50	117	<0.02	<0.02	192	0.3
H	7.55	159	<0.02	<0.02	280	0.36
I	7.70	246	<0.02	<0.02	403	0.23

n.a.: not analyzed.

Table S2. Analytical results: concentrations of DBPs in units of µg/L (data reprinted from Redondo-Hasselerharm et al. [1]). MBAA, monobromoacetic acid; DCAA, dichloroacetic Acid; BCAA, bromochloroacetic acid; DBAA, dibromoacetic acid; TCAA, trichloroacetic acid; BDCAA, bromodichloroacetic acid; DBCAA, dibromochloroacetic acid; TBAA,tribromoacetic acid; 1,1,1-TCP, 1,1,1- trichloropropanone(acetone); DCAN, dichloroacetonitrile; BCAN, bromochloroacetonitrile; DBAN, dibromoacetonitrile.

MCAA (monochloroacetic acid) was <2 µg/L, MIAA (monoiodoacetic acid) and DIAA (diiodoacetic acid) were <0.5 µg/L, chloropicrin and 1,1-dichloropropanone(acetone) were <0.1 µg/L and are not listed in the table.

**Tap water (postcode)**	**MBAA**	**DCAA**	**BCAA**	**DBAA**	**TCAA**	**BDCAA**	**DBCAA**	**TBAA**	**ΣHAAs**	**1,1,1TCP**	**DCAN**	**BCAN**	**DBAN**	**ΣHANs**
08001	1.7	< 0.5	1.0	< 0.5	< 0.5	< 0.5	< 0.5	< 0.5	**2.7**	<0.1	0.2	0.4	2.9	**3.5**
08002	1.2	< 0.5	2.2	11.3	< 0.5	< 0.5	1.7	3.9	**20.3**	<0.1	0.3	0.5	3.2	**1.2**
08003	0.7	< 0.5	< 0.5	3.8	< 0.5	< 0.5	< 0.5	< 0.5	**4.5**	<0.1	< 0.1	0.2	1.7	**1.9**
08004	1.1	< 0.5	1.0	9.0	< 0.5	< 0.5	< 0.5	< 0.5	**11.1**	<0.1	< 0.1	0.7	4.3	**5.0**
08005	1.7	< 0.5	< 0.5	< 0.5	1.3	< 0.5	< 0.5	< 0.5	**3.0**	<0.1	0.4	0.7	3.9	**5.0**
08006	< 0.5	11.9	2.6	< 0.5	21.8	1.8	< 0.5	< 0.5	**38.1**	1.1	1.6	0.5	1.1	**3.2**
08007	1.0	< 0.5	0.7	7.5	< 0.5	< 0.5	< 0.5	0.9	**10.1**	<0.1	< 0.1	0.4	2.7	**3.1**
08008	0.9	< 0.5	2.1	8.5	< 0.5	< 0.5	1.0	2.5	**15.0**	<0.1	0.3	0.4	2.5	**3.2**
08009	0.5	< 0.5	< 0.5	6.1	< 0.5	< 0.5	1.5	3.6	**11.8**	<0.1	< 0.1	0.5	2.8	**3.3**
08010	< 0.5	< 0.5	< 0.5	4.4	< 0.5	< 0.5	0.7	1.7	**6.7**	<0.1	< 0.1	0.6	3.4	**4.0**
08011	1.0	< 0.5	0.9	8.0	< 0.5	< 0.5	1.3	2.5	**13.7**	<0.1	< 0.1	0.4	2.4	**2.8**
08012	< 0.5	< 0.5	< 0.5	0.7	1.1	< 0.5	0.7	1.8	**4.3**	<0.1	0.4	0.4	2.8	**3.3**

Tap water (postcode)	MBAA	DCAA	BCAA	DBAA	TCAA	BDCAA	DBCAA	TBAA	ΣHAAs	1,1,1TCP	DCAN	BCAN	DBAN	ΣHANs
08013	< 0.5	12.9	4.9	1.6	16.3	1.8	< 0.5	< 0.5	**37.5**	1.2	2.3	0.8	0.4	**3.5**
08014	0.9	< 0.5	0.9	8.7	< 0.5	< 0.5	2.4	5.1	**18.0**	<0.1	0.3	0.5	2.7	**3.5**
08015	< 0.5	< 0.5	< 0.5	4.6	< 0.5	< 0.5	< 0.5	< 0.5	**4.6**	<0.1	< 0.1	0.2	2.2	**2.4**
08016	< 0.5	14.2	1.9	< 0.5	18.2	2.6	< 0.5	< 0.5	**36.9**	1.3	2.1	0.5	0.1	**2.7**
08017	1.4	3.8	1.1	5.2	5.2	< 0.5	< 0.5	< 0.5	**15.7**	<0.1	1.1	0.8	2.8	**4.7**
08018	< 0.5	< 0.5	< 0.5	< 0.5	< 0.5	< 0.5	< 0.5	< 0.5	**-**	<0.1	< 0.1	0.2	1.9	**2.1**
08019	1.2	< 0.5	2.0	12.2	0.8	< 0.5	1.0	2.5	**19.8**	<0.1	< 0.1	0.5	3.6	**4.2**
08020	1.5	< 0.5	1.2	7.8	< 0.5	< 0.5	< 0.5	< 0.5	**10.5**	<0.1	< 0.1	0.4	2.7	**3.1**
08021	< 0.5	9.8	1.8	< 0.5	22.5	3.7	< 0.5	< 0.5	**37.8**	1.5	2.6	0.6	0.1	**3.3**
08022	< 0.5	9.5	2.4	1.5	23.9	1.8	< 0.5	< 0.5	**39.1**	1.3	1.9	0.4	0.1	**2.4**
08023	< 0.5	6.4	1.5	< 0.5	20.1	1.6	< 0.5	< 0.5	**29.6**	1.2	1.8	0.5	0.3	**2.6**
08024	< 0.5	1.8	< 0.5	< 0.5	19.1	< 0.5	< 0.5	< 0.5	**20.9**	1.3	2.0	0.5	0.2	**2.7**
08025	< 0.5	11.3	1.7	< 0.5	21.8	1.6	< 0.5	< 0.5	**36.4**	2.0	2.5	0.5	0.1	**3.1**
08026	< 0.5	15.1	< 0.5	< 0.5	17.6	1.5	< 0.5	< 0.5	**34.2**	1.1	2.0	0.4	< 0.1	**2.4**
08027	0.6	10.8	2.9	0.8	19.1	2.2	0.9	0.7	**38.0**	0.9	1.4	0.6	0.8	**2.8**
08028	0.7	< 0.5	< 0.5	8.9	< 0.5	< 0.5	1.3	2.9	**13.7**	0.5	< 0.1	0.6	3.2	**3.8**
08029	1.0	< 0.5	< 0.5	7.7	< 0.5	< 0.5	2.0	4.8	**15.5**	<0.1	< 0.1	0.5	3.4	**3.9**
08030	1.2	< 0.5	1.4	8.8	< 0.5	< 0.5	2.7	4.0	**18.1**	<0.1	< 0.1	0.9	3.7	**4.6**

**Tap water (postcode)**	**MBA A**	**DCA A**	**BCA A**	**DBA A**	**TCA A**	**BDCA A**	**DBCA A**	**TBA A**	**ΣHAA s**	**1,1,1TC P**	**DCA N**	**BCA N**	**DBA N**	**ΣHAN s**
08031	< 0.5	11.1	< 0.5	< 0.5	22.5	2.4	< 0.5	< 0.5	**36.0**	1.2	2.1	0.4	0.1	**2.6**
08032	< 0.5	12.4	1.9	< 0.5	22.5	2.4	< 0.5	< 0.5	**39.2**	1.8	2.4	0.5	< 0.1	**2.9**
08033	0.9	4.0	2.2	7.3	6.6	1.6	2.9	2.9	**28.4**	<0.1	0.6	0.8	2.6	**4.0**
08034	0.9	< 0.5	< 0.5	6.2	< 0.5	< 0.5	< 0.5	< 0.5	**7.1**	<0.1	< 0.1	0.5	3.3	**3.8**
08035	< 0.5	5.6	< 0.5	< 0.5	16.3	2.0	< 0.5	< 0.5	**23.9**	1.3	2.1	0.5	< 0.1	**2.6**
08036	0.6	< 0.5	0.6	3.8	< 0.5	< 0.5	< 0.5	< 0.5	**5.0**	<0.1	< 0.1	0.2	2.1	**2.3**
08037	0.8	< 0.5	1.1	8.4	< 0.5	< 0.5	1.7	3.7	**15.8**	<0.1	< 0.1	0.6	3.8	**4.4**
08038	0.5	< 0.5	< 0.5	2.6	< 0.5	< 0.5	< 0.5	< 0.5	**3.1**	<0.1	< 0.1	0.1	1.2	**1.3**
08039	1.1	< 0.5	< 0.5	13.9	< 0.5	0.6	3.4	6.6	**25.6**	<0.1	< 0.1	0.8	4.2	**5.0**
08040	1.0	< 0.5	1.0	7.5	< 0.5	< 0.5	1.3	3.5	**14.3**	<0.1	< 0.1	0.4	3.2	**3.6**
08041	< 0.5	6.6	1.8	0.7	13.4	1.4	0.7	0.8	**25.4**	0.6	1.3	0.5	0.8	**2.6**
08042	< 0.5	9.4	1.7	< 0.5	17.5	1.7	< 0.5	< 0.5	**30.3**	<0.1	1.7	0.5	<0.1	**2.2**
**Activated carbon filters**	**MBA A**	**DCA A**	**BCA A**	**DBA A**	**TCA A**	**BDCA A**	**DBCA A**	**TBA A**	**ΣHAA s**	**1,1,1TC P**	**DCA N**	**BCA N**	**DBA N**	**ΣHAN s**
08001	< 0.5	< 0.5	< 0.5	0.8	< 0.5	0.1	0.8	1.6	**3.3**	< 0.1	< 0.1	0.2	1.1	**1.3**
08006	< 0.5	6.0	1.4	< 0.5	7.8	1.0	< 0.5	< 0.5	**16.2**	< 0.1	0.3	< 0.1	0.1	**0.4**
08008	0.9	< 0.5	< 0.5	6.0	< 0.5	< 0.5	1.4	3.3	**11.6**	< 0.1	< 0.1	0.2	1.6	**1.8**
08013	< 0.5	8.7	< 0.5	< 0.5	6.3	1.1	< 0.5	< 0.5	**16.1**	0.3	0.6	0.1	< 0.1	**0.7**

**Tap water (postcode)**	**MBA A**	**DCA A**	**BCA A**	**DBA A**	**TCA A**	**BDCA A**	**DBCA A**	**TBA A**	**ΣHAA s**	**1,1,1TC P**	**DCA N**	**BCA N**	**DBA N**	**ΣHAN s**
08017	< 0.5	0.8	< 0.5	0.7	< 0.5	< 0.5	< 0.5	< 0.5	**1.5**	< 0.1	< 0.1	< 0.1	< 0.1	**-**
08028	0.7	< 0.5	0.8	6.3	< 0.5	< 0.5	0.9	2.2	**10.9**	< 0.1	< 0.1	< 0.1	0.4	**0.4**
**Reverse Osmosis**	**MBA A**	**DCA A**	**BCA A**	**DBA A**	**TCA A**	**BDCA A**	**DBCA A**	**TBA A**	**ΣHAA s**	**1,1,1TC P**	**DCA N**	**BCA N**	**DBA N**	**ΣHAN s**
08002	< 0.5	< 0.5	< 0.5	< 0.5	< 0.5	< 0.5	< 0.5	< 0.5	**-**	< 0.1	< 0.1	< 0.1	< 0.1	**-**
08018	< 0.5	< 0.5	< 0.5	< 0.5	< 0.5	< 0.5	< 0.5	< 0.5	**-**	< 0.1	< 0.1	< 0.1	< 0.1	**-**
08019	< 0.5	< 0.5	< 0.5	< 0.5	< 0.5	< 0.5	< 0.5	< 0.5	**-**	< 0.1	< 0.1	< 0.1	< 0.1	**-**
08024	< 0.5	< 0.5	< 0.5	< 0.5	< 0.5	< 0.5	< 0.5	< 0.5	**-**	< 0.1	< 0.1	< 0.1	< 0.1	**-**
08029	< 0.5	< 0.5	< 0.5	< 0.5	< 0.5	< 0.5	< 0.5	< 0.5	**-**	< 0.1	< 0.1	< 0.1	< 0.1	**-**
**Bottled water**	**MBA A**	**DCA A**	**BCA A**	**DBA A**	**TCA A**	**BDCA A**	**DBCA A**	**TBA A**	**ΣHAA s**	**1,1,1TC P**	**DCA N**	**BCA N**	**DBA N**	**ΣHAN s**
A-I	< 0.5	< 0.5	< 0.5	< 0.5	< 0.5	< 0.5	< 0.5	< 0.5	**-**	< 0.1	< 0.1	< 0.1	< 0.1	**-**

Text S1. Additional information on the experimental method for direct neurotoxicity measurement with unenriched water

Aliquots of the sampled water were filtered, aliquoted and frozen until their use. One aliquot of each sample was supplemented with DEMEM/F12 powder medium (Gibco BRL, Life Technologies, Paisley, UK) and pH was adjusted to 7.4. Then, samples were filtered with 0,45 µm sterile syringe microfilters (Millipore Merk, Burlington, MA, USA) and stored at –20°C. The same process was performed with fresh miliQ water (Merk Millipore, Billerica, MA, USA). For all the different experiments, samples were supplemented with 10% FBS and 1% penicillin/streptomycin (Gibco BRL, Life Technologies, Paisley, UK), with the exception of the experiment of neurite length where samples were complemented with 1 % FBS, 1 % PS and 10 µM of all-trans retinoic acid (RA, from Sigma Aldrich (Merck KGaA, Darmstadt, Germany) to induce their differentiation. DMEM/F12 media served as negative control (Gibco BRL, Life Technologies, Paisley, UK).

The human SH-SY5Y neuroblastoma cell line was purchased from American Type Culture Collection (Manassas, VA, USA). Cells were maintained in complete culture medium consisting of Dulbecco's modified Eagle's medium (DMEM)/F12 pH 7.4 (Gibco BRL, Life Technologies, Paisley, UK) supplemented with 10% heat-inactivated fetal bovine serum (FBS) and 1% penicillin/streptomycin (PS, Gibco BRL, Life Technologies, Paisley, UK). The cells were maintained in a humidified incubator with 5% CO_2 and 95% air at 37°C. The medium was refreshed every 2-3 days.

Cell viability was quantified using the metabolic dye 3-[4,5-dimethylthiazol-2-yl]−2,5-diphenyltetrazolium bromide (MTT, Sigma, St. Louis, MO, USA). When cultures were confluent (70–80%), cells were seeded in 48-well tissue culture plates at density of $2×10^4$ cells/well in complete medium. After 24 h of cell attachment, cells were exposed to tap water samples media for 24 h. MTT was added to the cells 3 h prior to the end of the experiment at a final concentration of 0.5 mg mL^{-1}. During that time, MTT was reduced to produce a dark blue formazan product. Formazan production was measured after removal of the medium and extraction with DMSO by the change in absorbance at 560 nm using a microplate reader (BioTek Power Wave XS). The viability results were expressed as a percentage of control.

The Lactate Dehydrogenase (LDH) cytotoxicity assay was performed with the help of LDH assay kit to confirm cell damage. The experiments were carried out following the manufacturer's protocol (CyQUANT™ LDH Cytotoxicity Assay (C20300) manufactured by Thermo Fisher Scientific Inc.). SH-SY5Y cells were grown in a 48-well plate at density of $2×10^4$ cells/well in complete medium and were incubated over night with 5% CO_2 and 95% air at 37°C. The cells were incubated with tap water samples media for 24 hours. To determinate maximum LDH activity, 20 µL of 10X Lysis buffer was added to no treated well for 45 min before elapsed time. Plates were then centrifuged at 400 x g for five minutes and 50 µL of cell

supernatant was transferred to a new 96-well assay plate in triplicate wells. Then, 50 µL of reaction mixture solution was added to each well. Plates were incubated with gentle shaking on an orbital shaker for 30 min at room temperature and 50 µL of stop solution was added to each sample well. The absorbance was read at 490 nm and 680 nm using a microplate reader (BioTek, Power Wave XS, USA). To determine LDH activity, the 680-nm absorbance value (background) was subtracted from the 490-nm absorbance before calculation of % cytotoxicity using the spontaneous LDH activity of the solvent control and the maximum LDH activity of the positive control in relation to the LDH activity of the water sample:

$$\%\ \text{Cytotoxicity} = \frac{(\text{LDH activity of water sample} - \text{spontaneous LDH activity})}{(\text{maximum LDH activity} - \text{spontaneous LDH activity})} \times 100$$

Cell death was assessed by the uptake of the fluorescent exclusion dye propidium iodide. This method relies upon the fact that an intact nuclear membrane has low permeability for propidium iodide. However, when cell integrity becomes compromised, it gains access to the nucleus where it complexes with DNA rendering the nucleus highly fluorescent. SH-SY5Y cells (2×10^4 cells/well) on 48-well plates were incubated with tap water samples media for 24 h, then stained with propidium iodide (PI, Sigma, St. Louis, MO, USA) solution at final concentration of 20 µg/mL for 5 min in the dark. Immediately thereafter microscope images were obtained using a fluorescence microscopy Olympus BX61 (Olympus America Inc., NY, USA) with excitation 540 nm and emission 620 nm. Cell death was determined as the percentage of red fluorescent cells per total cells counted.

To determine the effect of tap water samples on neuronal morphological differentiation of SH-SY5Y cells, tap water samples media was supplemented with 1% PS, 1% FBS and with 10 µM all-trans retinoic acid (Sigma Aldrich). After incubation for 3 days, the cells were fixed in 4% paraformaldehyde in phosphate-buffered saline (PBS) for 3 min and washed with PBS. The morphological changes in the cells were observed under a phase-contrast microscope Olympus BX61 (Olympus America Inc., NY, USA). Briefly, the neurite length was determined by measuring the total length of the outgrowing neurites per soma using Image J software (v. 1.53p). Those cells whose cell body diameters longer than twice of the diameter of cell body were considered as neurite-bearing cells. The average of neurites length was determined by counting at least 200 cells in each sample.

Statistical analysis was performed using the software Statistical Package for the Social Sciences (SPSS v.27). The homogeneity of variances was analysed with Levene's test. If variances were homogenous, ANOVA was then used followed by Bonferroni's test in order to analyse all dose groups simultaneously. The Kruskal-Wallis test was used when variances were not homogeneous. Significance was set at $p < 0.05$.

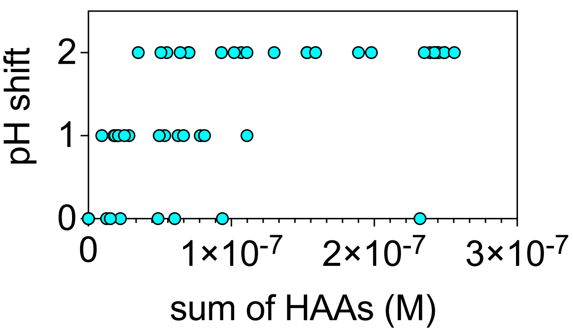

Figure S1. pH shift towards lower pH appears to be related to the sum of molar concentrations of haloacetic acids (HAAs).

Figure S2. Concentration-response curves in AREc32 (selected examples).

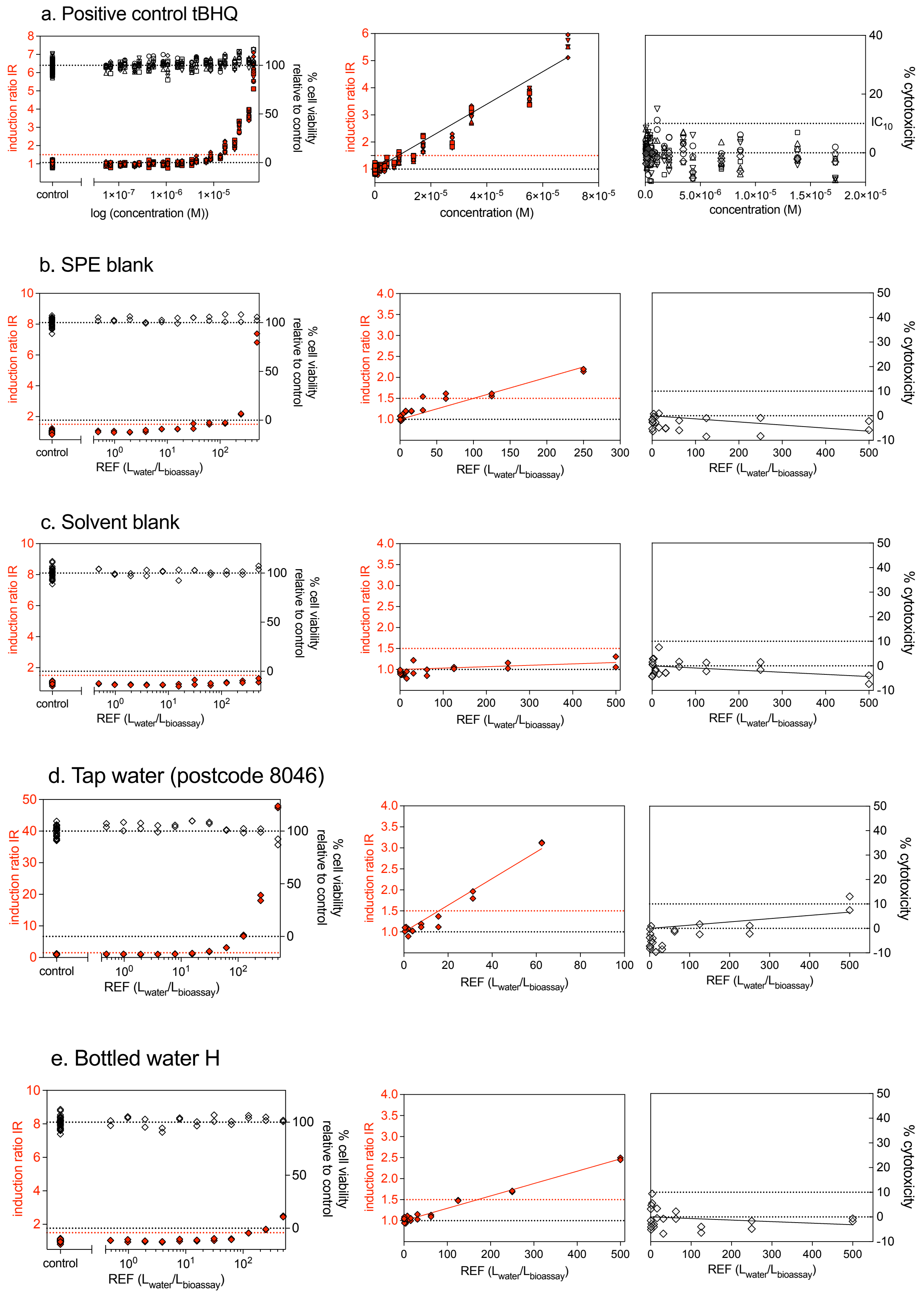

Table S3. Inhibitory concentration IC_{10} for 10 % reduction of cell viability in the AREc32 cell line and effect concentration $EC_{IR1.5}$ for activation of oxidative stress response of the water samples. SE = standard error, calculated with error propagation from the SE of the slope of the CRC [2]. The LOD was IR(LOD) = $\mu_{IR,\ unexposed\ cells} + (3 \times \sigma_{IR,\ unexposed\ cells}) = 1.0 + 3 \times 0.09 = 1.29$.

**Sample**	**Cytotoxicity**		**Activation of oxidative stress response**	
**Positive control**	**IC_{10} (M)**	**SE**	**$EC_{IR1.5}$ (M)**	**SE**
tBHQ	>2.00E-5		8.34E-06	1.51E-07
**Negative control**	**IC_{10} (REF)**	**SE**	**$EC_{IR1.5}$ (REF)**	**SE**
SPE Blank 1	>500		113	9
SPE Blank 2	>300		80.4	4.7
SPE Blank 3	>500		284	18
SPE Blank 4	>500		424	58
SPE Blank 5	>500		>500	
**Tap water (postcode)**	**IC_{10} (REF)**	**SE**	**$EC_{IR1.5}$ (REF)**	**SE**
08001	>500		19.4	0.7
08002	>500		21.4	0.7
08003	>500		37.8	1.4
08004	>500		17.0	0.7
08005	>500		23.9	1.1
08006	194	20	11.3	0.5
08007	359	72	23.8	0.9
08008	373	93	28.8	0.8
08009	397	121	19.7	1.1
08010	442	142	25.1	1.4
08011	>500		21.0	0.5
08012	325	52	16.3	0.6
08013	339	51	47.7	3.4
08014	128	12	19.7	0.9
08015	257	41	24.8	1.0
08016	107	9	26.9	1.7
08017	>500		21.5	1.3

**Sample**	**Cytotoxicity**		**Activation of oxidative stress response**	
08018	>500		27.4	1.0
08019	196	24	13.0	0.4
08020	247	36	21.2	1.1
08021	>500		140.7	41.5
08022	214	30	12.0	0.5
08023	126	18	13.7	0.6
08024	82	20	22.5	0.9
08025	180	17	11.6	0.5
08026	124	9	36.3	2.6
08027	156	12	13.9	0.4
08028	>500		40.2	0.9
08029	450	153	19.2	0.9
08030	111	13	14.1	0.8
08031	174	18	9.0	0.3
08032	225	30	12.2	0.5
08033	193	29	18.7	0.8
08034	400	88	22.8	0.7
08035	67	8	21.2	0.8
08036	264	35	28.0	0.6
08037	>500		19.1	0.7
08038	>500		39.5	1.9
08039	61	4	13.3	1.0
08040	>500		22.8	0.6
08041	>500		14.8	0.7
08042	237	34	17.5	1.0
**Activated carbon filters**	**IC_{10} (REF)**	**SE**	**$EC_{IR1.5}$ (REF)**	**SE**
08001	170	21	38.2	1.5
08006	205	36	22.5	1.1
08008	>500		30.2	1.2
08013	>500		12.2	0.4

**Sample**	**Cytotoxicity**		**Activation of oxidative stress response**	
08017	>500		76.9	4.0
08028	259	43	27.9	1.1
**Reverse Osmosis**	**IC_{10} (REF)**	**SE**	**$EC_{IR1.5}$ (REF)**	**SE**
08002	>500		178	11
08018	>500		253	21
08019	>500		161	7
08024	>500		159	8
08029	>500		223	13
**Bottled water**	**IC_{10} (REF)**	**SE**	**$EC_{IR1.5}$ (REF)**	**SE**
A	>500		283	23
B	>500		158	12
C	>500		>500	
D	246	37	153	19
E	>500		472	73
F	>500		186	8
G	>500		281	18
H	>500		170	5
I	>500		334	30

Text S2. Results the direct neurotoxicity measurement with unenriched water.

Cell viability assay did not show significant (p>0.05) changes on treated cells with tap water samples media over 24 h, respect to control (Fig. S3). In addition, tap water samples media treatment also showed no significance differences in LDH leakage after 24 h of exposition, respect to control (Fig. S3). These results suggest that contaminants present in tap water samples had not high enough concentration to cause effect on cell viability and cytotoxicity on SH-SY5Y cells.

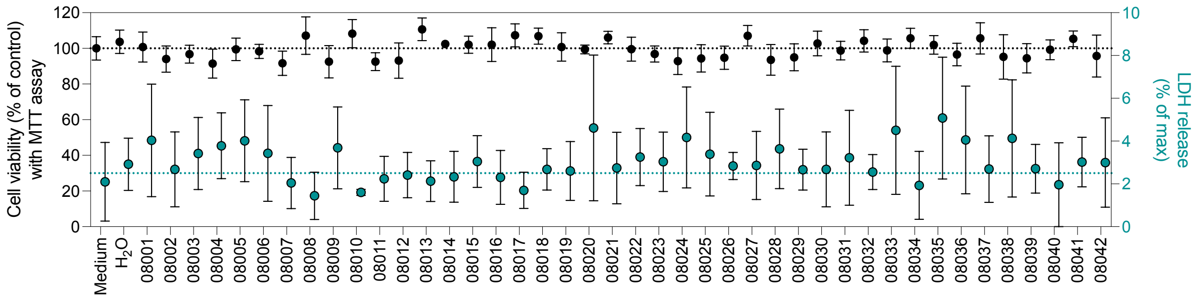

Figure S3. Cytotoxicity of SH-SY5Y dosed directly with water. SH-SY5Y cell viability quantified with MTT (left y-axis) and LDH leakage (right y-axis) measured after 24h of exposure to tap water samples media. Cell viability and LDH leakage data are expressed as the mean ± SD of four independent experiments. Significant differences relative to the control (medium DEMEM/F12) were analyzed by one-way ANOVA followed by Bonferroni's post hoc test: *p < 0.05 versus control.

Staining with PI did not show significant (p>0.05) increase of membrane damage or apoptotic cells on treated cells with tap water samples media over 24 h, respect to control (Fig. S2A). Only treated cells with 15 % of DMSO (positive control) showed a significant diminution (p < 0.05) respect to control. These results suggest that DBPs and other contaminants present in tap water samples were not sufficiently concentrations induce apoptotic cell death or damage in membrane on SH-SY5Y cells.

Differentiation of SH-SY5Y with 10 µM RA in presence of tap water samples media did not show any significant (p>0.05) differences on average of neurite length after 72 h of exposition, respect to control (Fig. 2SB). These results suggest that contaminants present in tap water samples had not enough potency to disrupt neuronal differentiation of SH-SY5Y cells and the growing neurite length.

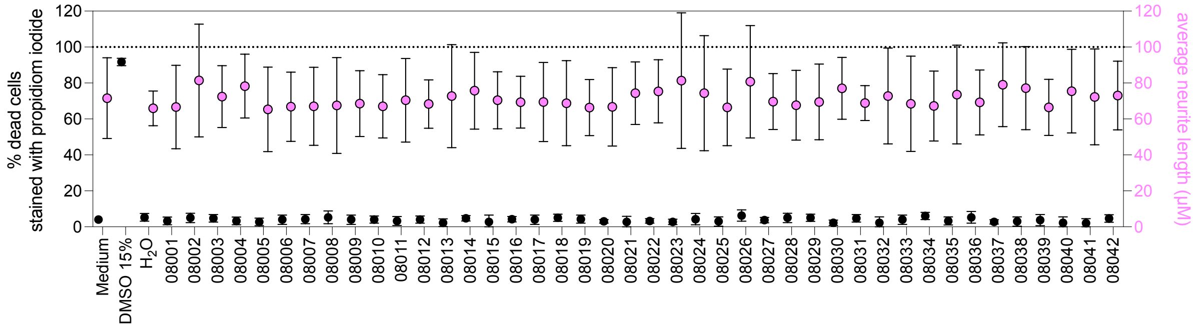

Figure S4. SH-SY5Y cells neurite outgrowth inhibition assessment with directly dosed water**.** SH-SY5Y cells were stained with PI (left y-axis) after 24 h of exposition to tap water samples media. The cells were also differentiated (right y-axis) with 10 µM retinoic acid in tap water samples media over 72 h. The percentage of the positive cells stain, respect to the total cells, and the average of neurite length data are expressed as the mean ± SD of four independent experiments. Significant differences relative to the control (DEMEM/F12) were analyzed by one-way ANOVA followed by Bonferroni's post hoc test: *p < 0.05 versus control.

Figure S5. Concentration-response curves in the neurotoxicity assay dosed with enriched water extracts. (a) positive control narciclasine, (b), (c) SPE blanks, (d) to (j) tap water, (k) water filtered with pitcher-type activated carbon (AC) filter (l)-(m) tap water treated with reverse osmosis (RO), (n)-(o) bottled water. Only this selection of samples was measured.

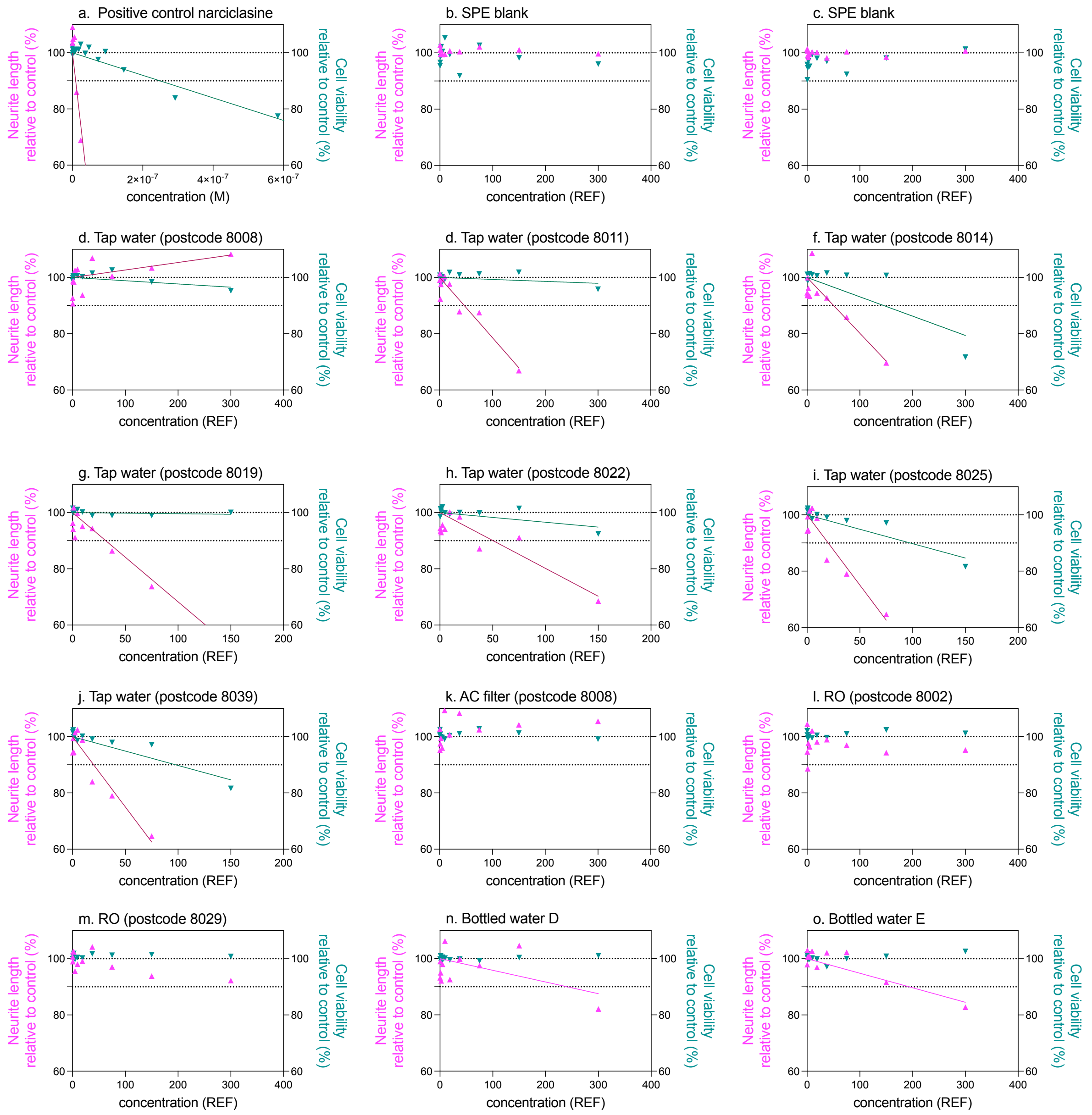

Figure S6. Effect of acidity of some of the samples in the neurotoxicity assay. Phase-contrast images of the differentiated SH-SY5Y cells show the cell bodies as dark grey areas and the neurites as pink lines connecting the cells in (a) unexposed cells, (b) cells exposed to tap water 08019, (c) artefacts observed in tap water 08008 due to cell debris on the background.

a. Control

b. Exposed cells without cell debris

c. Exposed cells with cell debris

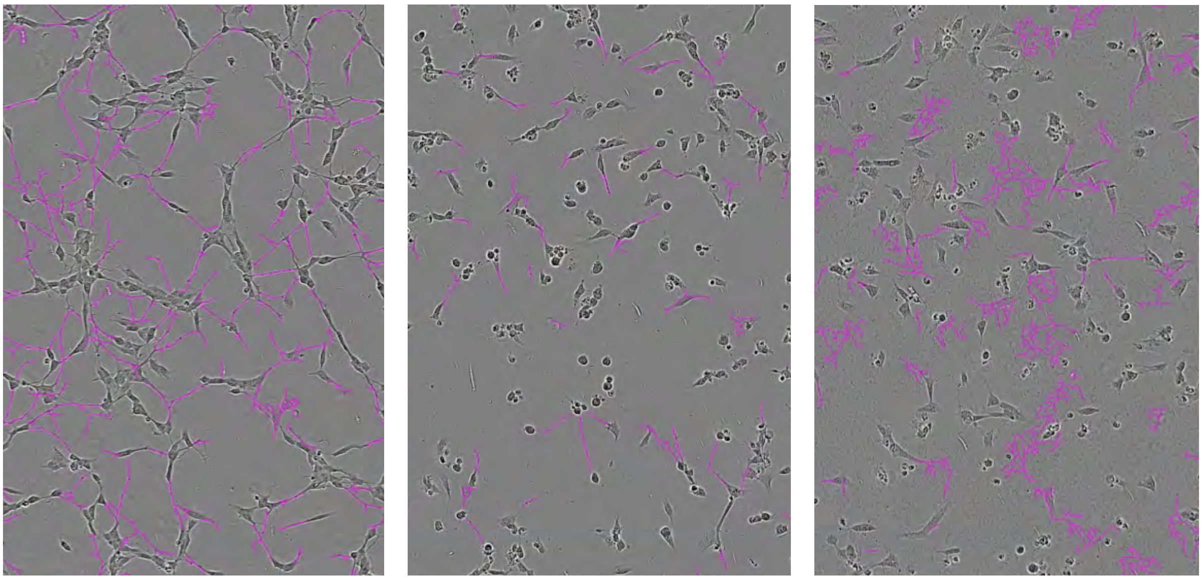

Table S4. Inhibitory concentration IC_{10} for 10 % reduction of cell viability in the neuronal cell line SH-SY5Y and effect concentration EC_{10} for reduction of neurite length by 10% for selected samples.

	Cytotoxicity		Neurite outgrowth inhibition	
**Positive control**	**IC_{10} (M)**	**SE**	**EC_{10} (M)**	**SE**
Narciclasine	2.49E-07	2.08E-08	9.17E-09	3.49E-09
**Negative control**	**IC_{10} (REF)**	**SE**	**EC_{10} (REF)**	
SPE Blank	>300		>300	
SPE Blank	>300		>300	
**Tap water (postcode)**	**IC_{10} (REF)**	**SE**	**EC_{10} (REF)**	**SE**
08008	>300		>150[a]	
08011	>300		46.8	4.2
08014	145.6	30.3	50.5	7.4
08019	>300		31.5	2.2
08022	290.7	92.5	50.4	7.5
08025	97.8	12.8	20.0	2.0
08039	189.9	55.9	46.1	11.8
**Activated carbon filters**	**IC_{10} (REF)**		**EC_{10} (REF)**	
08008 (AC)	>300		>150[b]	
**Reverse osmosis**	**IC_{10} (REF)**		**EC_{10} (REF)**	
08002 (RO)	>300		>300	
08029 (RO)	>300		>300	
**Bottled water**	**IC_{10} (REF)**		**EC_{10} (REF)**	**SE**
D	>300		193.6	32.0
E	>300		240.6	102.2

[a]Artefacts due to cell debris observed in neurite outgrowth measurement at a REF of 300; no cell debris observed below REF 300.

[b]Artefacts due to cell debris observed in neurite outgrowth measurement at REF 150 and 300; no cell debris observed below REF 150.

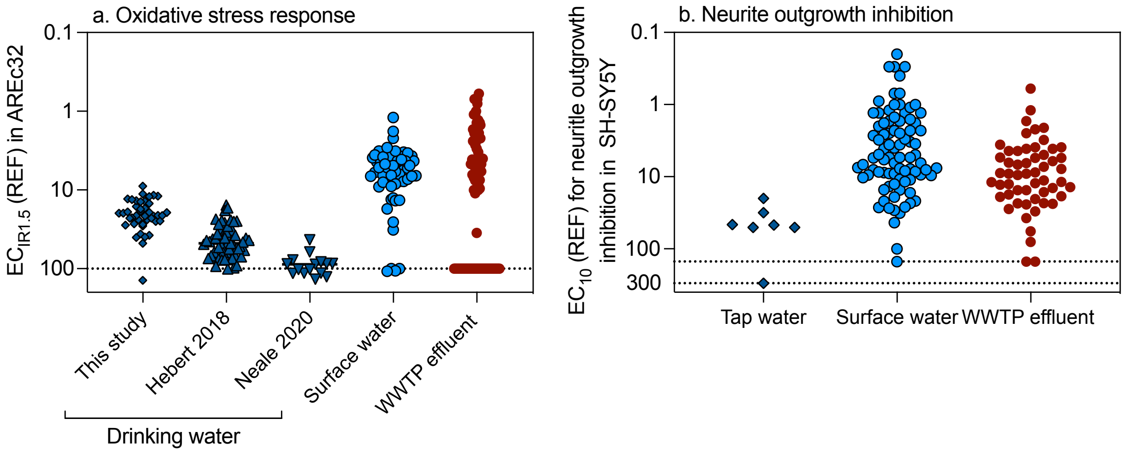

Figure S7. Comparison of the effect concentrations of tap water effect data of this study with literature of drinking water and other water types. (a) Oxidative stress response detected with AREc32 compared with drinking water from literature (Hebert *et al.* 2018 [3] and Neale *et al.* 2020 [4]), surface water during rain events [5] and wastewater treatment plant effluent (WWTP) [5]. (a) EC_{10} in the neurite outgrowth inhibition assay compared with EC_{10} of surface water during rain events [5] and wastewater treatment plant effluent (WWTP) [5].

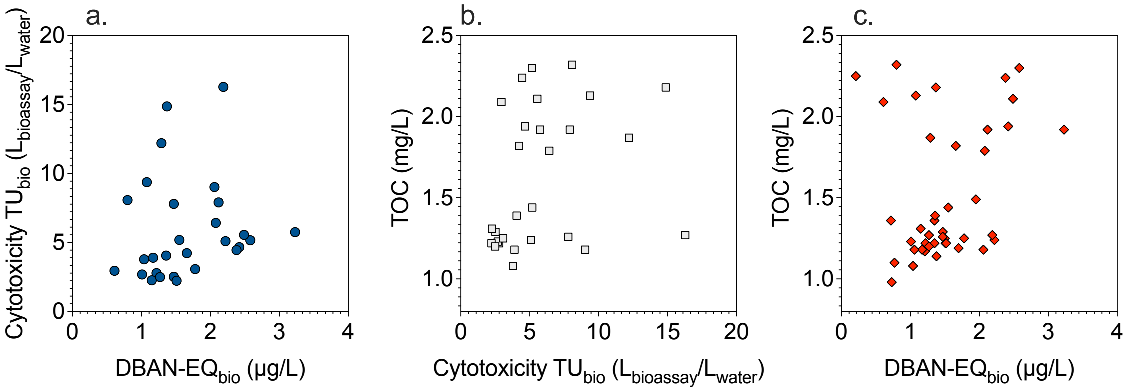

Figure S8. Comparison of cytotoxicity, oxidative stress response and total organic carbon (TOC) of the tap water; (a) Oxidative stress response expresses as DBAN-EQ_{bio} compared to cytotoxicity toxic units TU_{bio}; (b) TU_{bio} compared with TOC; (c) DBAN-EQ_{bio} compared to TOC.

Table S5. Effect concentrations $EC_{IR1.5}$ and for the detected chemicals in AREc32. Relative effect potency of $REP_i = EC_{IR1.5}(DBAN)/ EC_{IR1.5}(i)$. Data from Stalter et al. [6].

Disinfection byproduct	Abbreviation	$EC_{IR1.5}$ in AREc32	relative effect potency REP
Chloroacetic acid	MCAA	$2.65 \cdot 10^{-4}$	$5.50 \cdot 10^{-4}$
Bromoacetic acid	MBAA	$5.19 \cdot 10^{-6}$	$2.81 \cdot 10^{-2}$
Dichloroacetic Acid	DCAA	$6.03 \cdot 10^{-6}$	$2.42 \cdot 10^{-5}$
Bromochloroacetic acid	BCAA	$1.41 \cdot 10^{-4}$	$1.03 \cdot 10^{-3}$
Dibromoacetic acid	DBAA	$1.15 \cdot 10^{-4}$	$1.26 \cdot 10^{-3}$
Trichloroacetic acid	TCAA	>0.02	-
bromodichloroacetic acid	BDCAA	$2.00 \cdot 10^{-3}$	$7.29 \cdot 10^{-6}$
Dibromochloroacetic acid	DBCAA	$4.90 \cdot 10^{-3}$	$2.98 \cdot 10^{-5}$
Tribromoacetic acid	TBAA	$4.41 \cdot 10^{-4}$	$3.30 \cdot 10^{-4}$
1,1,1- Trichloropropanone	1,1,1-TCP	$1.54 \cdot 10^{-5}$	$9.51 \cdot 10^{-3}$
Dichloroacetonitrile	DCAN	$7.72 \cdot 10^{-6}$	$1.89 \cdot 10^{-2}$
Bromochloroacetonitrile	BCAN	$2.23 \cdot 10^{-6}$	$6.52 \cdot 10^{-2}$
Dibromoacetonitrile	DBAN	$1.46 \cdot 10^{-7}$	1

Table S6. Iceberg modeling: comparison of the bioanalytical equivalent concentrations DBAN-EQ_{bio} with the DBAN-EQ_{chem}, and the contribution of the individual quantified DBPs *i,* DBAN -EQ_{bio}(*i*). Units of DBAN-EQ are μg/L.

Tap water (postcode)	DBAN-EQ_{chem} (1,1,1TCP)	DBAN-EQ_{chem} (ΣHAA)	DBAN-EQ_{chem} (ΣHAN)	DBAN-EQ_{chem} (ΣHAA; HAN; 1,1,1TCP)	DBAN-EQ_{bio}	%explained by 1,1,1TCP	%explained by ΣHAA	%explained by ΣHAN
08001	0	0.069	2.91	2.98	1.50	0%	4.6%	195%
08002	0	0.065	3.28	3.35	1.35	0%	4.8%	243%
08003	0	0.033	1.68	1.72	0.77	0%	4.2%	219%
08004	0	0.056	4.40	4.45	1.70	0%	3.3%	258%
08005	0	0.068	4.00	4.07	1.21	0%	5.6%	330%
08006	0.013	0.004	1.21	1.23	2.58	0.5%	0.1%	47%
08007	0	0.050	2.78	2.83	1.22	0%	4.1%	229%
08008	0	0.049	2.57	2.62	1.01	0%	4.9%	255%
08009	0	0.029	2.88	2.91	1.47	0%	2.0%	196%
08010	0	0.005	3.47	3.47	1.15	0%	0.5%	301%
08011	0	0.051	2.39	2.44	1.38	0%	3.7%	174%
08012	0	0.001	2.84	2.84	1.78	0%	0.1%	160%
08013	0.014	0.008	0.55	0.57	0.61	2.2%	1.4%	91%
08014	0	0.048	2.75	2.80	1.47	0%	3.3%	187%
08015	0	0.005	2.16	2.17	1.17	0%	0.5%	185%
08016	0.015	0.003	0.19	0.21	1.08	1.4%	0.3%	18%

Tap water (postcode)	DBAN-EQ$_{chem}$ (1,1,1TCP)	DBAN-EQ$_{chem}$ (ΣHAA)	DBAN-EQ$_{chem}$ (ΣHAN)	DBAN-EQ$_{chem}$ (ΣHAA; HAN; 1,1,1TCP)	DBAN-EQ$_{bio}$	%explained by 1,1,1TCP	%explained by ΣHAA	%explained by ΣHAN
08017	0	0.064	2.92	2.99	1.35	0%	4.7%	217%
08018	0	0	1.95	1.95	1.06	0%	0%	184%
08019	0	0.067	3.65	3.72	2.22	0%	3.0%	164%
08020	0	0.070	2.76	2.83	1.36	0%	5.1%	202%
08021	0.017	0.003	0.29	0.31	0.21	8.4%	1.3%	139%
08022	0.016	0.005	0.23	0.25	2.42	0.6%	0.2%	10%
08023	0.014	0.002	0.38	0.40	2.12	0.7%	0.1%	18%
08024	0.015	0	0.35	0.37	1.29	1.1%	0%	27%
08025	0.024	0.003	0.18	0.21	2.49	1.0%	0.1%	7%
08026	0.013	0.001	0.10	0.12	0.80	1.7%	0.1%	13%
08027	0.010	0.029	0.94	0.98	2.08	0.5%	1.4%	45%
08028	0.005	0.038	3.26	3.31	0.72	0.8%	5.3%	453%
08029	0	0.050	3.40	3.45	1.51	0%	3.3%	225%
08030	0	0.061	3.79	3.85	2.06	0%	3.0%	184%
08031	0.014	0.001	0.19	0.20	3.23	0.4%	0.0%	6%
08032	0.021	0.003	0.12	0.14	2.38	0.9%	0.1%	5%
08033	0	0.048	2.70	2.75	1.55	0%	3.1%	174%
08034	0	0.043	3.34	3.38	1.27	0%	3.4%	262%

Tap water (postcode)	DBAN-EQ$_{chem}$ (1,1,1TCP)	DBAN-EQ$_{chem}$ (ΣHAA)	DBAN-EQ$_{chem}$ (ΣHAN)	DBAN-EQ$_{chem}$ (ΣHAA; HAN; 1,1,1TCP)	DBAN-EQ$_{bio}$	%explained by 1,1,1TCP	%explained by ΣHAA	%explained by ΣHAN
08035	0.015	0	0.11	0.13	1.37	1.1%	0.0%	8%
08036	0	0.029	2.09	2.12	1.04	0%	2.8%	202%
08037	0	0.045	3.82	3.87	1.52	0%	3.0%	252%
08038	0	0.023	1.24	1.26	0.73	0%	3.1%	169%
08039	0	0.063	4.24	4.30	2.19	0%	2.9%	194%
08040	0	0.051	3.21	3.26	1.27	0%	4.0%	253%
08041	0.007	0.003	0.89	0.90	1.95	0.4%	0.2%	45%
08042	0	0.002	0.10	0.10	1.66	0%	0.1%	6%

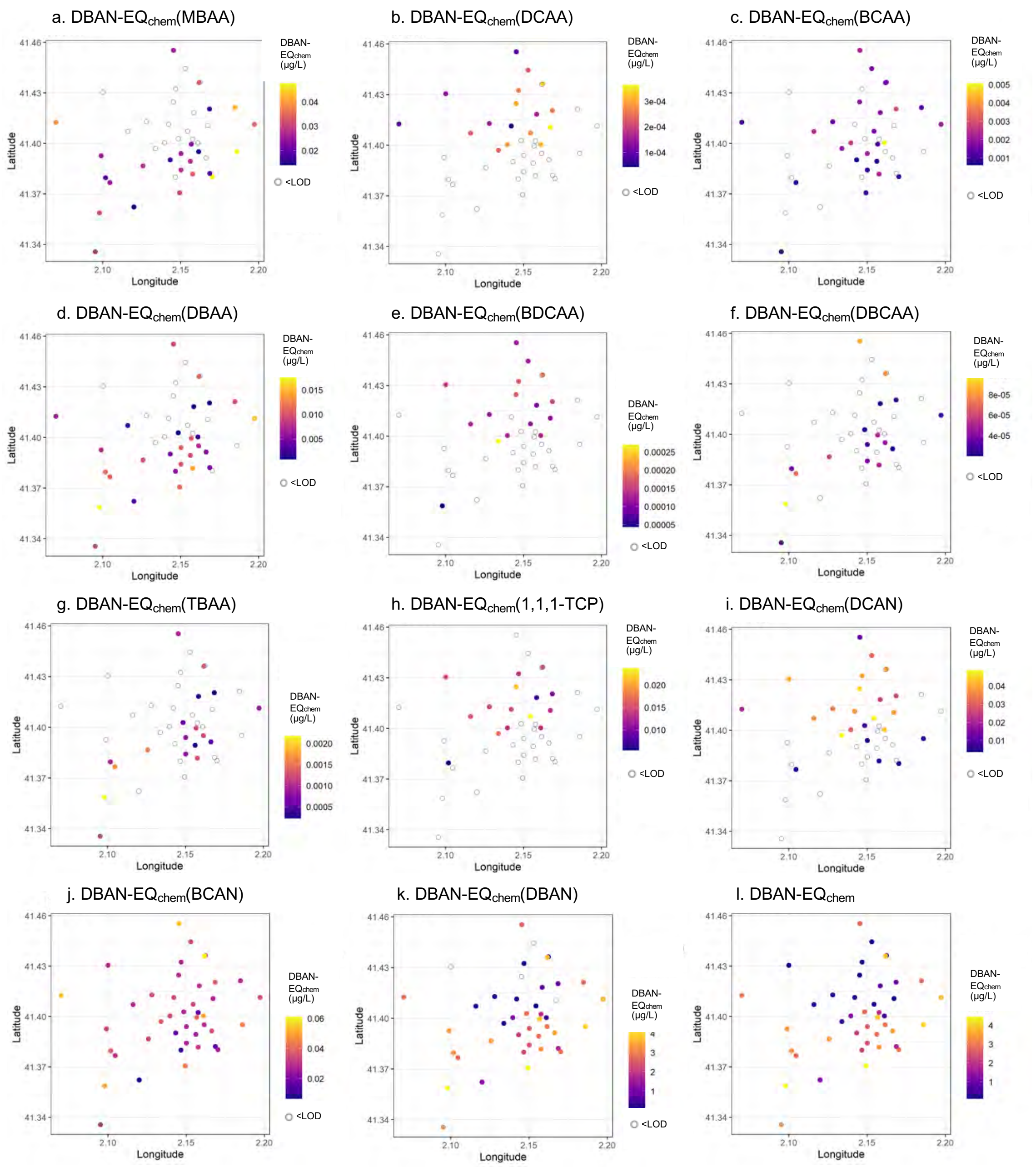

Figure S9. Spatial distribution of the DBAN-EQ$_{chem}$(*i*) of (a) MBAA, (b) DCAA, (c) BCAA, (d) DBAA, (e) BDCAA, (f) DBCAA, (g) TBAA, (h) 1,1,1TCP, (i) DCAN, (j) BCAN, (k) DBAN and (l) the sum of DBAN-EQ$_{chem}$(*i*), DBAN-EQ$_{chem}$ (eq. 2) in units of µg/L.

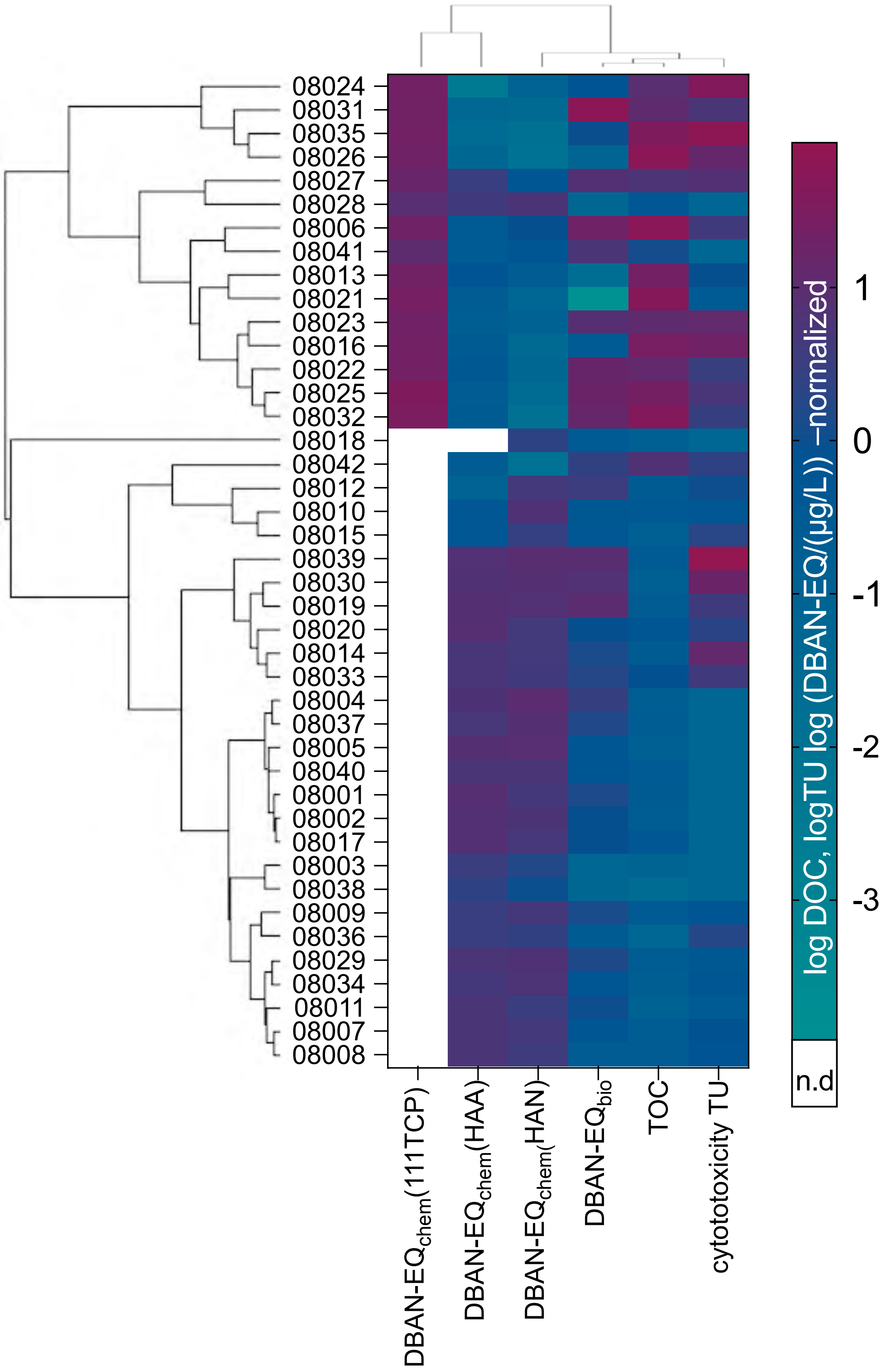

Figure S10. Hierarchical clustering after scaling of measured effects expressed as DBAN-EQ$_{bio}$, predicted effects DBAN-EQ$_{chem}$(ΣHAA), DBAN-EQ$_{chem}$(ΣHAN), DBAN-EQ$_{chem}$(1,1,1-TCP), cytotoxicity toxic units TU and total organic carbon (TOC).

Table S7. Percent reduction of total organic carbon (TOC), sum of concentrations of haloacetic acids (HAA) and haloacetonitriles (HAN) and reduction of predicted mixture effects CAA-EQ_{chem} from HAA and HAN as well as measured mixture effect CAA-EQ_{bio}. Data from Tables S1, S2 and S3.

**Post code**	**Reduction TOC (%)**	**Reduction sum concen-tration of HAA**	**Reduction sum concen-tration of HAN**	**Reduction DBAN-EQ_{chem} (HAA) (%)**	**Reduction DBAN-EQ_{chem} (HAN) (%)**	**Reduction DBAN-EQ_{chem} (%)**	**Reduction DBAN-EQ_{bio} (%)**
**Pitcher-type activated carbon filters**							
**08001**	-0.6%	29.4%	66.0%	98.1%	62.7%	63.5%	49.3%
**08006**	48.3%	57.0%	86.0%	46.5%	93.3%	93.1%	50.0%
**08008**	2.6%	27.6%	47.4%	10.6%	38.1%	37.6%	4.8%
**08013**	-0.7%	54.5%	77.9%	95.1%	94.7%	94.7%	-290%
**08017**	-38.3%	90.7%	100%	98.7%	100%	100%	72.1%
**08028**	17.5%	18.0%	90.5%	5.5%	88.5%	87.5%	-44.3%
**Reverse Osmosis**							
**08002**	57.7%	100%	100%	100%	100%	100%	88.0%
**08018**	77.1%	not detected	100%	not detected	100%	100%	89.1%
**08019**	84.9%	100%	100%	100%	100%	100%	91.9%
**08024**	76.6%	100%	100%	100%	100%	100%	85.8%
**08029**	75.3%	100%	100%	100%	100%	100%	91.4%

References

1. Redondo-Hasselerharm PE, Cserbik D, Flores C, Farré MJ, Sanchís J, Alcolea JA *et al* Insights to estimate exposure to regulated and non-regulated disinfection by-products in drinking water. Journal of Exposure Science & Environmental Epidemiology 2022; doi 10.1038/s41370-022-00453-6.

2. Escher B, Neale PA, Villeneuve D The advantages of linear concentration-response curves for *in vitro* bioassays with environmental samples. Environ Toxicol Chem 2018; 37: 2273–2280.

3. Hebert A, Feliers C, Lecarpentier C, Neale P, Schlichting R, Thibert S *et al* Bioanalytical assessment of adaptive stress responses in drinking water as a tool to differentiate between micropollutants and disinfection by-products. Water Res 2018; 132: 340-349.

4. Neale P, Feliers C, Glauch L, Lecarpentier C, Schlichting R, Thibert S *et al* Application of in vitro bioassays for water quality monitoring in three drinking water treatment plants

using different treatment processes including biological treatment, nanofiltration and ozonation coupled with disinfection. Environ Sci: Water Res Technol 2020; 6: 2444-2453.

5. Lee J, Schlichting R, König M, Scholz S, Krauss M, Escher BI Monitoring Mixture Effects of Neurotoxicants in Surface Water and Wastewater Treatment Plant Effluents with Neurite Outgrowth Inhibition in SH-SY5Y Cells. ACS Environmental Au 2022; doi 10.1021/acsenvironau.2c00026. 10.1021/acsenvironau.1022c00026.

6. Stalter D, O'Malley E, von Gunten U, Escher BI Fingerprinting the reactive toxicity pathways of 50 drinking water disinfection by-products. Water Res 2016; 91: 19-30.
